# A brain-penetrant triazolopyrimidine enhances microtubule-stability, reduces axonal dysfunction and decreases tau pathology in a mouse tauopathy model

**DOI:** 10.1186/s13024-018-0291-3

**Published:** 2018-11-07

**Authors:** Bin Zhang, Yuemang Yao, Anne-Sophie Cornec, Killian Oukoloff, Michael J. James, Pyry Koivula, John Q. Trojanowski, Amos B. Smith, Virginia M.-Y. Lee, Carlo Ballatore, Kurt R. Brunden

**Affiliations:** 1 Center for Neurodegenerative Disease Research, Perelman School of Medicine, University of Pennsylvania, 3600 Spruce St, Philadelphia, PA 19104 USA; 2 Department of Chemistry, School of Arts and Sciences, University of Pennsylvania, 231 South 34th St, Philadelphia, PA 19104-6323 USA; 3 Skaggs School of Pharmacy and Pharmaceutical Sciences, University of California, San Diego, 9500 Gilman Dr, La Jolla, CA 92093 USA

**Keywords:** Microtubule, Tauopathy, Therapeutic, Alzheimer’s disease

## Abstract

**Background:**

Alzheimer’s disease (AD) and related tauopathies are neurodegenerative diseases that are characterized by the presence of insoluble inclusions of the protein tau within brain neurons and often glia. Tau is normally found associated with axonal microtubules (MTs) in the brain, and in tauopathies this MT binding is diminished due to tau hyperphosphorylation. As MTs play a critical role in the movement of cellular constituents within neurons via axonal transport, it is likely that the dissociation of tau from MTs alters MT structure and axonal transport, and there is evidence of this in tauopathy mouse models as well as in AD brain. We previously demonstrated that different natural products which stabilize MTs by interacting with β-tubulin at the taxane binding site provide significant benefit in transgenic mouse models of tauopathy. More recently, we have reported on a series of MT-stabilizing triazolopyrimidines (TPDs), which interact with β-tubulin at the vinblastine binding site, that exhibit favorable properties including brain penetration and oral bioavailability. Here, we have examined a prototype TPD example, CNDR-51657, in a secondary prevention study utilizing aged tau transgenic mice.

**Methods:**

9-Month old female PS19 mice with a low amount of existing tau pathology received twice-weekly administration of vehicle, or 3 or 10 mg/kg of CNDR-51657, for 3 months. Mice were examined in the Barnes maze at the end of the dosing period, and brain tissue and optic nerves were examined immunohistochemically or biochemically for changes in MT density, axonal dystrophy, and tau pathology. Mice were also assessed for changes in organ weights and blood cell numbers.

**Results:**

CNDR-51657 caused a significant amelioration of the MT deficit and axonal dystrophy observed in vehicle-treated aged PS19 mice. Moreover, PS19 mice receiving CNDR-51657 had significantly lower tau pathology, with a trend toward improved Barnes maze performance. Importantly, no adverse effects were observed in the compound-treated mice, including no change in white blood cell counts as is often observed in cancer patients receiving high doses of MT-stabilizing drugs.

**Conclusions:**

A brain-penetrant MT-stabilizing TPD can safely correct MT and axonal deficits in an established mouse model of tauopathy, resulting in reduced tau pathology.

**Electronic supplementary material:**

The online version of this article (10.1186/s13024-018-0291-3) contains supplementary material, which is available to authorized users.

## Background

The tauopathies are neurodegenerative diseases characterized by the presence of insoluble inclusions of the tau protein within brain neurons and often glia. These tau accumulations are referred to as neurofibrillary tangles (NFTs) when found in the neuronal soma and neuropil threads (NTs) when found in dendritic processes [1, 2]. AD is the most prevalent tauopathy, where the hallmark pathologies are NFT, NT and neuritic plaque-associated tau inclusions, as well as senile plaques comprised of amyloid β peptides [3]. In contrast, neuronal and/or glial tau inclusions are the primary pathology in other tauopathies that include progressive supranuclear palsy (PSP), corticobasal degeneration (CBD), Pick’s disease and other frontotemporal lobar degenerative (FTLD) conditions [1]. There is a strong correlation between tau pathological burden in the brain and cognitive decline in AD [4–6], a finding bolstered by recent tau positron emission tomography imaging studies in AD [7, 8] and FTLD due to tau pathology [9, 10], suggesting that it is the development of abundant tau inclusions that ultimately leads to the neurodegeneration observed in AD and the other tauopathies. That tau mutations lead to familial cases of FTLD with NFTs and NTs [11, 12] further confirms that misfolded tau oligomers and/or inclusions are sufficient to cause neurodegeneration.

Tau is normally found associated with axonal MTs in the brain, and in tauopathies this MT binding is diminished due to tau hyperphosphorylation [13–15], facilitating tau deposition into the fibrillar accumulations that comprise NFTs and NTs. Tau binding to MTs is thought to reduce MT dynamicity, particularly at the more labile distal portions of MTs [16, 17], thereby providing increased stability to this region of axonal MTs either directly [18] and/or through inhibition of MT-severing enzymes [19, 20]. As MTs play a critical role in the movement of vesicles, mitochondria and other cellular constituents within neurons via axonal transport [21], it is likely that the dissociation of tau from MTs in tauopathies alters both MT structure and axonal transport, although the observation of axonal transport deficits in other neurodegenerative diseases with neuronal protein inclusions (e.g., Parkinson’s disease and amyotrophic lateral sclerosis) suggests that inclusions themselves may affect MT structure and/or function [22]. There is compelling evidence of MT abnormalities in neuronal [23] and transgenic (Tg) mouse models [24–27] of tauopathy, with the latter showing decreased MT density, increased MT dynamicity, and slowed axonal transport. MT deficits have also been observed in AD brain [28–30], and it is thus likely that altered MT structure and function contributes to the neurodegenerative processes in tauopathies [31]. In fact, studies from our laboratories and others have revealed that treatment of tau Tg mice with brain-penetrant MT-stabilizing natural products such as epothilone D (EpoD) and dictyostatin improves a number of CNS outcomes, with enhanced MT density, axonal transport, neuron survival, and cognitive performance with a reduction of tau pathology [24, 25, 27, 32]. Notably, EpoD proved to be particularly safe and efficacious in tauopathy models, and EpoD (BMS-241027) subsequently advanced to Phase 1b testing in AD patients (ClinicalTrials.gov identifier NCT01492374), where it was found to be safe in a 9-week trial.

**Fig. 1:**
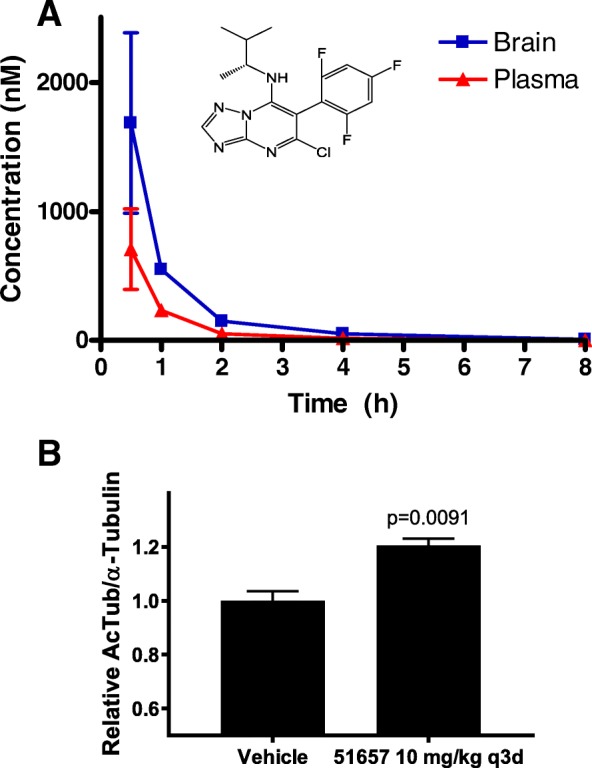
PK and pharmacodynamic profiling of 51657. **a** Plasma and brain levels of 51657 (structure inset) after a 5 mg/kg i.p. dose. Brain levels exceed plasma levels at all times, with terminal plasma and brain T_1/2_ values of 1–1.5 h. Error bars represent SEM, with *n* = 3 per time point. **b** Levels of brain AcTub in WT mice 3 days after the last dose of vehicle or 10 mg/kg of 51657. Error bars represent SEM of *n* = 3 per treatment, with an unpaired t-test used to determine significance of compound effect

Given the therapeutic potential of brain-penetrant MT-stabilizing compounds, we have recently evaluated non-naturally occurring small molecule MT-stabilizing agents, with the goal of identifying alternative and potentially improved candidates for development as disease-modifying drugs for AD and other neurodegenerative conditions. These efforts led to the characterization of a series of brain-penetrant TPD and phenylpyrimidine (PPD) MT-modulating molecules [33–35] that, when compared to EpoD and dictyostatin, exhibit several favorable features including oral bioavailability, lack of P-glycoprotein (Pgp) interaction and ease of synthesis. Notably, the mechanism of action of these MT-active small molecules is believed to be unique and distinct from that of EpoD, dictyostatin and other MT taxane-site binders, as binding [36, 37] and X-ray crystallography [38] studies revealed that TPDs interact with β-tubulin at a site that largely overlaps with the vinblastine binding site. An evaluation of representative PPD and TPD examples revealed an unexpected divergence of MT-directed activity of these molecules, in which all active PPDs and a large proportion of active TPDs demonstrated a bell-shaped concentration-response profile when markers of stabilized MTs (i.e., acetylated and detyrosinated α-tubulin [39], or AcTub and GluTub, respectively) were quantified in cellular assays [35]. Moreover, the PPD and TPD molecules that elicited this unusual concentration-response caused MT disruption at higher concentrations, as visualized by immunocytochemistry, with an associated proteasome-mediated degradation of cellular tubulin [35]. In contrast, a subset of the TPD molecules (referred to as TPD+ compounds) elicited linear concentration-dependent increases in stable MT markers and in cellular MT mass in both transformed cells and primary neuron cultures. Moreover, a prototype TPD+ molecule (CNDR-51657; hereafter 51657, structure in Fig. 1a) was shown to rescue neuron cultures from axonal damage resulting from MT-destabilization [35]. In addition, 51657 was shown to increase brain AcTub in wild-type (WT) mice after a single administration [35].

Here, we have selected 51657 as a prototype TPD+ compound for more complete in vivo characterization, including efficacy testing in the PS19 tau Tg mouse model of tauopathy [40]. We reveal that 51657 provided benefit in PS19 mice, including increased MT density, reduced axonal dystrophy and a significant reduction of brain tau pathology, features that closely resemble the salutary effects previously obtained with EpoD. These data demonstrate that a MT-stabilizing agent that interacts with MTs at a site distinct from the taxane/epothilone binding site can provide benefits in a neurodegenerative disease model that are comparable to those observed with taxane-site binders. Thus, TPD+ molecules hold promise as potential therapeutic agents for AD and other neurodegenerative diseases.

## Methods

### Compound synthesis

The synthesis of 51657 was conducted at the 0.5 g scale following procedures described previously [35]. The spectroscopic properties of the compound were identical to those reported in the literature. In addition, single crystal x-ray diffraction analysis of the final compound was conducted (see Supplemental Information).

### ADR-RES cytotoxicity assay

ADR-RES cells (NCI) were maintained in RPMI medium (Mediatech) containing 10% FBS, 2 mM L-glutamine, and 1% penicillin/streptomycin (complete RPMI) at 37 °C in 5% CO_2_. For compound testing, cells were dissociated with trypsin/EDTA and plated at a density of 3000 cells/well in black 96-well clear-bottom plates (Perkin-Elmer) in 0.1 ml of complete RPMI medium, followed 24 h later by the addition of paclitaxel or 51657 diluted from 10 mM DMSO stock solutions that were diluted into complete RPMI medium (0.1 ml total added to existing medium; final compound concentration of 1 μM). In addition, wells were also treated with 0.1 ml of vehicle alone (final concentration of 0.01% DMSO on cells). Cells were maintained at 37 °C in 5% CO_2_ and at 72 h after compound addition, 20 μl of Alamar Blue cell viability reagent (Invitrogen) was added to the wells and allowed to incubate for 4 h at 37 °C in 5% CO_2_ followed by measurement in a SpectraMax M5 plate reader with excitation of 550 nm and emission of 590 nm with a cutoff of 570 nm. A set of vehicle-treated wells were treated with digitonin (final concentration of 0.5%) at the time of compound addition to kill cells and elicit the minimal Alamar Blue signal. The percent cell viability was calculated as 100 × (Test-Digitonin)/(Vehicle-Digitonin).

### Microsomal metabolism of 51657

Pooled human and mouse liver microsomes (Corning Life Sciences) were utilized at a concentration of 1 mg/ml with a NADPH regenerating system as per vendor instructions. Compound (51657) was added at 1 μM in the absence or presence of CYP450 inhibitors, and aliquots of the reaction mixture were removed at 10 min intervals for 60 min. Acetonitrile was added to the sampled reactions at 3:1 (*v*/v) and the mixtures were vortexed and centrifuged, with the supernatant subjected to LC-MS/MS analysis as previously described [35].

### Mouse studies

All methods utilizing mice were first submitted and approved by the University of Pennsylvania Institutional Animal Care and Use Committee (IACUC).

### Analysis of plasma and brain compound concentrations

Test compound was administered to 2–4 month old CD-1 or B6SJL mice, with both female and male mice utilized but sexes were not mixed within experimental groups. For standard single time-point brain and plasma determinations, groups of mice (*n* = 3) were injected intraperitoneally (i.p.) with a single dose of 5 mg/kg compound dissolved in DMSO. For pharmacokinetic analysis, groups of mice (n = 3) were sacrificed at various times points after i.p. dosing of 5 mg/kg of compound. Whole brain hemispheres were homogenized in 10 mM ammonium acetate, pH 5.7 (50%, *w*/*v*), using a hand-held sonic homogenizer. Plasma was obtained from blood collected in 0.5 M EDTA solution and centrifuged for 10 min at 4,500 × g at 4 °C. The analysis of compound concentrations in plasma and brain homogenates was as previously described [35].

### Brain AcTub determinations

CD-1 female mice (n = 3; 2–3 months of age) received three i.p. injections of 10 mg/kg of 51657 spaced 72 h apart. After 72 h following the third injection, mice were euthanized by an IACUC-approved protocol and cortices were dissected from each brain and placed immediately in ice-cold RIPA buffer (50 mM Tris, 150 mM NaCl, 5 mM EDTA, 0.5% sodium deoxycholate, 1% NP-40, 0.1% SDS, pH 8.0) containing protease -inhibitor cocktail (Sigma Aldrich), 1 mM phenylmethylsulfonyl fluoride (PMSF) (Sigma Aldrich), and 3 μM trichostatin A (Sigma Aldrich). Tissue was homogenized with a hand-held battery operated pestle motor mixer and then sonicated to complete the lysis. Samples were centrifuged at 100,000 × g for 30 min at 4 °C and supernatant was transferred to a new Eppendorf tube. Remaining pellets were re-suspended in RIPA buffer and homogenized, sonicated, and centrifuged again, as before. Supernatant from the second centrifugation step was pooled with that from first spin. Samples were assessed for protein concentration by bicinchoninic acid (BCA) assay (Thermo Fisher Scientific) and enzyme-linked immunosorbent assay (ELISA) analysis of acetyl- and alpha-tubulin levels was performed, as previously described [35, 41].

### 51657 Treatment of PS19 Tg mice

PS19 mice [40] express a transgene encoding the human T34 tau isoform (1N4R) containing the P301S mutation found in inherited FTLD-tau [42]. Groups of 9-month old female PS19 mice (B6C3/F1 background as described [40]) were administered twice-weekly i.p. injections of 3 mg/kg or 10 mg/kg of 51657 at a volume of 2 μl/g body weight, or vehicle only (9% DMSO/91% corn oil), for a total of 12 weeks. An additional group of age-matched non-transgenic female littermates were treated with vehicle as above. Mice entered into the study in 4 separate cohorts spaced over 4 months, with each cohort having all groups represented such that the final group size of all treatment arms reached *n* = 12. All mice were monitored for signs of abnormal behavior or distress, and were weighed weekly to monitor body weight. After 11 weeks of dosing, the mice from 3 of 4 study cohorts underwent Barnes maze testing as described below. After sacrifice by an IACUC-approved protocol, blood was collected from 3 of 4 study cohorts for complete blood cell counts, as described [25]. Similarly, the optic nerve (ON), which harbors tau pathology together with retinal ganglia cells in these mice [25], was recovered from 3 of 4 study cohorts for transmission electron microscopy (EM) analysis of axonal dystrophy and MT density. Brains were collected from all study mice for biochemical and immunohistochemical analyses, and organ weights were recorded to assess compound tolerability.

### Body weights, organ weights and complete blood cell counts

Study mice were weighed once-weekly during the course of the dosing period. Upon sacrifice and perfusion, key organs were collected and weights determined. Blood samples from a subset of the study WT and PS19 mice, as indicated in the figure legend, were sent to an outside vendor (7th Wave Laboratories, St. Louis, MO) for complete blood cell analyses.

### ON axonal dystrophy and MT density analyses

EM was performed on cross sections of ON from vehicle- or 51657-treated WT and PS19 mice to assess MT density and axonal dystrophy, as previously described [27, 32].

### Immunoblot analysis of insoluble brain tau

Combined cortex and hippocampus samples (~ 40–50 mg) from frozen hemispheres of vehicle- and 51657-treated PS19 mice were homogenized in 0.2 ml of RAB high salt buffer (0.1 M MES, 1 mM EGTA, 0.5 mM MgSO_4_, 0.75 M NaCl, 0.02 M NaF, pH 7.0), and the homogenates were centrifuged at 100,000 × g for 30 min at 4 °C. The resulting pellet was resuspended in 0.2 ml RAB buffer and centrifuged as above, followed by another resuspension in 0.3 ml of RAB buffer followed by centrifugation. The remaining pellet was resuspended in 0.2 ml of RIPA buffer (50 mM Tris, 150 mM NaCl, 0.1% SDS, 0.5% sodium deoxycholate, 1% NP40 and 5 mM EDTA), followed by centrifugation as above. This pellet was resuspended in 0.1 ml of 2% SDS and sonicated, followed by centrifugation at 100,000 × g for 30 min at 22 °C. The SDS pellet was resuspended in 0.1 ml of 2% SDS followed by sonication and centrifugation, and the resulting supernatant was combined with the first SDS supernatant. This combined SDS supernatant fraction was utilized for SDS-PAGE analysis and immunoblotting as previously described [35], using a rabbit polyclonal antibody recognizing total tau (17205 [43]; developed in-house, RRID:AB_2315435, used at 1:2000 dilution of sera in Li-Cor blocking buffer), a rabbit polyclonal antibody recognizing tau containing an acetyl modification at lysine residue 280 (TauAcK280; developed in-house [44], used at 1:2000 dilution of sera in Li-Cor blocking buffer) or the AT8 monoclonal antibody (ThermoFisher) that recognizes tau that is phosphorylated at serine residue 202 and/or threonine residue 205 (1:2000 dilution of sera in Li-Cor blocking buffer). Immunoblots were imaged using an Odyssey IR imaging system (Li-Cor) and relative protein amounts were quantified from the immunoblots using ImageStudio software (Li-Cor). Because the majority of the total protein, including housekeeping proteins, are extracted into the RAB-soluble fraction, the RAB-insoluble, SDS-soluble brain samples from each PS19 mouse were loaded onto SDS-PAGE gels at equal protein amounts based on the corresponding RAB-soluble protein concentration. More specifically, RAB-insoluble samples, which were all solubilized in equal volumes of SDS as described above, were prepared for SDS-PAGE such that the amounts loaded corresponded to 0.25 mg/ml of the RAB-soluble fraction. For example, if the RAB-soluble protein concentration was 2 mg/ml, the RAB-insoluble sample was diluted 8-fold for SDS-PAGE analysis. To provide further normalization accuracy, corresponding samples of the RAB-soluble fractions diluted to 0.25 mg/ml were loaded onto separate SDS-PAGE gels and blotted for the housekeeping protein, GAPDH. The immunoblot densitometric values for the tau species from the RAB-insoluble samples for each mouse were then normalized to the corresponding GAPDH densitometric value from the RAB-soluble sample (see Additional file 1: Figure S6 for representative blot images). All samples for immunoblot analyses were coded so as to mask the sample identification throughout the immunoblot procedure, including during densitometric quantification of the tau and GAPDH bands.

### Tau ELISA

The RAB-soluble fractions from brain homogenates of vehicle- and 51657-treated PS19 mice (see above) were assessed for total tau utilizing a sandwich ELISA essentially as previously described [27], with volumes of the samples adjusted based on total protein content in the RAB-soluble fraction as determined by BCA assay.

### AT8 immunohistochemistry

Study mice were perfused with PBS (20 ml) after being deeply anesthetized using a protocol approved by the University of Pennsylvania IACUC. The brains were subsequently removed and one hemisphere from each mouse was processed as previously described [25, 27], with 6 μm thick paraffin-embedded sections prepared and stained with the AT8 antibody (1:2000 dilution) that recognizes tau phosphorylated at S202/T205 [45]. Immunostained sections that were masked to treatment group were imaged using a 4× microscopic objective. For analysis of hippocampal neurons, 3 matched brain sections (Bregma: − 2.20 to − 2.80) from vehicle- and 51657-treated PS19 mice were manually annotated around the entire hippocampus and entorhinal cortex using HALO (Indica Labs, Corrales, NM) software. Sections representing average AT8 staining intensity were thresholded to allow quantification of tau pathology in the hippocampal and cortical sections without contribution of background staining, and a common threshold was then applied to all sections. Quantification was conducted with the HALO software. The area of tau pathology within each annotated region was determined, and this was summed across the three individual sections from each mouse and divided by the sum of the total annotated area from the three sections to get the total % area with tau pathology. This value was multiplied by the average optical density (OD) of the tau pathology to yield the final “normalized AT8 area x OD”, and the sum of these values from the hippocampal and cortical assessments are reported.

### NeuN immunohistochemistry

Quantification of CA3 neurons was performed using NeuN antibody to label neuronal nuclei [46]. Staining was performed as noted above for AT8 staining, using a mouse anti-NeuN antibody (Millipore; 1:500). Two bregma levels (Bregma: − 1.82 and − 1.94) containing the CA3 region of the hippocampus were used for analyses. Slides were blinded and scanned using a Perkins Elmer Lamina slide scanner. ImageJ (NIH) was used for NeuN image analysis and quantification. Briefly, RGB TIFF images were converted to 8-bit images and then inverted. Max entropy auto-thresholding was used on all images and the CA3 region of the hippocampus was annotated manually using morphological landmarks in the mouse brain. Percent NeuN-positive area was then used as a readout for neuronal density, with the data decoded and compiled by an independent investigator.

### GFAP and Iba1 immunofluorescence

Paraffin-embedded sections (6 μm; Bregma − 2.5) from PS19 mouse brains receiving vehicle or 51657 (*n* = 3/group) as above were deparaffinized through a 5 min incubation in xylene followed by graded rehydration in ethanol solutions (100%, 95%, 80% and 75% for 1 min each). The sections then underwent antigen retrieval through addition of a citrate-based antigen unmasking solution (Vector Labs) followed by microwave treatment at 99 °C for 15 min. After washing of slides in 0.1 M Tris, the slides were stained with GFAP (rat 2.2B10 hybridoma supernatant; RRID:AB_2532994) and Iba1 (rabbit polyclonal; Dako) that were each diluted 1:1000 in 0.1 M Tris containing 2% fetal bovine serum, utilizing fluorescent anti-rat (AF594, 1:700; ThermoFisher) or anti-rabbit (AF488, 1:700; Wako) secondary antibodies. Images were then captured using a fluorescence microscope.

### Barnes maze analyses

Barnes Maze testing was performed as previously described [47] by an experimenter blinded to the treatment groups. Briefly, mice were handled for 3 days prior to Barnes Maze testing to get accustomed to the experimenter. Mice were habituated to the testing room for 30 min prior to testing each day. Mice were then placed in the center of Barnes Maze (San Diego Instruments, White 7001–0235) in the starting cylinder for 30 s. The starting cylinder was then removed and mice were allowed to explore the Barnes Maze for 2.5 min. If the mouse did not find the target box, the mouse was gently guided into the target box. The mice were allowed to remain in the target box for 1 min before returning them to their home cage. Two trials per mouse were performed each day with a 15-min inter-trial interval. Mice were tested for 4 consecutive days. The percent success was determined based on the mouse’s first encounter (≥ 2 s) with the target box, termed primary success. Primary measures were used because some mice would successfully locate the target box yet continue to explore the maze, a behavior which has been reported previously [48].

### Statistics

GraphPad Prism 7 was utilized for all statistical analyses. Comparisons between treatment groups consisted of unpaired t-tests when comparing two groups, or one-way ANOVA analyses with Tukey post-hoc analysis to compare between groups when comparing more than two groups. Grubb’s tests (GraphPad QuickCalc) were applied to the data to query for extreme outliers, and when found (as noted in figure legends) these outliers were removed from the data analysis.

## Results

### Pharmacokinetic (PK) and Pharmacodynamic properties of CNDR-51657

Among the TPD+ compounds, 51657 (Fig. 1a) was chosen as a prototype for full in vivo characterization. Prior analyses demonstrated that 51657 is orally bioavailable and has excellent brain penetration, with a brain-to-plasma (B/P) exposure ratio of ~ 2.7 at 1 h after i.p. dosing [35]. A more complete PK analysis of 51657 in WT mice confirmed that total brain exposure exceeded that in plasma, with terminal brain and plasma T_1/2_ values of ~ 1.0–1.5 h (Fig. 1a). Although the T_1/2_ of 51657 is somewhat short, we had previously demonstrated that a single 1 mg/kg i.p. dose increased WT mouse brain AcTub one day after administration, indicating target engagement and increased MT stability [35]. In further analyses, we found that a 10 mg/kg dose of 51657 administered once every 3–4 days over 7 days to WT mice resulted in elevated brain AcTub that persisted for 72 h after the final dosing (Fig. 1b). Given that the compound is eliminated from the brain relatively quickly, this prolonged MT activity suggests that brain MTs retain stability for an extended period after drug clearance. Alternatively, an active metabolite of 51657 may be formed with significantly longer brain retention than the parent compound. However, we have investigated the metabolism of 51657 in mouse and human microsomal studies and found that the molecule is metabolized by several CYP450 enzymes to release an inactive *N*-dealkylated derivative (Additional file 1: Figure S1A & B). An examination of mouse plasma and brain homogenates confirms the generation of high levels of this inactive metabolite after 51657 dosing (Additional file 1: Figure S1C), suggesting that the extended MT stabilization observed after 51657 dosing was not likely due to the formation of an active metabolite. The prolonged MT-stabilizing effect of 51657 could also result from an irreversible MT interaction. However, the crystal structure of a structurally-related TPD molecule bound to a MT [38], which we have determined is a TPD+ compound [49], reveals no evidence of a covalent interaction, suggesting it is unlikely that 51657 binds covalently to MTs. Finally, it is possible that a small fraction of 51657 remains non-covalently bound to brain MTs after the majority of drug clears from the brain, and that this amount is sufficient to impart increased AcTub. Regardless of the exact mechanism of this extended MT-stabilizing effect, our observations suggest that a long CNS residence time may not be required for meaningful MT stabilization, as was previously postulated for the MT-stabilizing agents EpoD and dictyostatin, both of which had very long brain T_1/2_ values [25, 50].

It has been reported that TPD molecules that are structurally related to 51657 are cytotoxic to Pgp-expressing cancer cell lines [38, 51], indicating that they are not Pgp substrates. This would differentiate such TPDs from paclitaxel and related MT-directed taxanes, as well as many other cancer drugs, which are ineffective against Pgp-expressing cells. We previously demonstrated that 51657 is not a competitive Pgp inhibitor [35], which indicates it does not interact with Pgp. To further verify an absence of Pgp binding, we examined the ability of 51657 to inhibit proliferation of Pgp-expressing ADR-RES cells [52] and compared its activity to paclitaxel, which is a Pgp substrate. As expected, 51657 (1 μM) promoted a significantly greater cytotoxicity than did the same concentration of paclitaxel (Additional file 1: Figure S2). This concentration of paclitaxel is at least two orders of magnitude greater than that required for cytotoxic activity in cell lines not expressing Pgp [53], and the greater effect of 51657 reveals that it is an effective cytotoxic agent for Pgp-expressing cells. Given the excellent brain exposure of 51657, these data would suggest that TPD+ compounds of this type might hold promise for the treatment of brain cancers such as astrocytomas or glioblastomas, as many anti-cancer agents have poor brain penetration due to Pgp efflux at the blood-brain barrier, and there is also evidence of Pgp-mediated drug resistance in some glioblastomas [54]. However, it is important to note that 51657 was present continuously in the cell culture medium in these cytotoxicity studies, and given the short plasma and brain half-life observed after dosing in mice, it is likely that frequent dosing of this compound would be required to elicit a meaningful anti-mitotic effect. In fact, as discussed further below, we observed no signs of cytotoxicity or anti-mitotic activity when 51657 was dosed twice-weekly in tau Tg mice, although this dosing schedule provided CNS benefit.

### Testing of CNDR-51657 in PS19 tau transgenic mice

Given the ability of 51657 to elicit a prolonged increase of AcTub in the WT mouse brain, we subsequently evaluated the compound for efficacy in the PS19 tau transgenic mouse model, which expresses human 1N4R tau harboring the P301S mutation found in inherited FTLD-tau [40]. We previously utilized this mouse model in both prevention and intervention studies to demonstrate the efficacy of EpoD [25, 27] and dictyostatin [32]. In these prior studies, only male PS19 mice were utilized because they develop tau pathology more rapidly than female PS19 mice and mixing of age-matched PS19 mice of both sexes results in unacceptably high variability in the amount of tau pathology that can mask treatment effects. Unpublished work from our laboratories revealed that female PS19 mice will also develop appreciable tau pathology, albeit with a 5–6-month delay relative to male PS19 mice. Moreover, young (2–3 month) female PS19 mice can develop tau pathology to an extent comparable to that observed in age-matched male PS19 mice when synthetic tau fibril “seeds” are introduced into the brain to initiate the formation of tau pathology [55, 56]. Because female mice can be group-housed to reduce study costs, we opted to examine 51657 in aged female PS19 mice. Groups of 9-month old female PS19 mice (*n* = 12/group) received vehicle, or 3 mg/kg or 10 mg/kg of 51657, twice-weekly (i.p.) for a total duration of 3 months. In addition, a group of age-matched non-transgenic female littermates received twice-weekly administration of vehicle. We anticipated that the 9-month old female PS19 mice would be roughly comparable to 3–4-month old male PS19 mice with regard to the extent of brain tau pathology, with the latter showing a low but detectable tau inclusion burden [25, 56]. Thus, the study was designed to be a secondary prevention assessment of 51657 efficacy, similar to our prior study in which EpoD was shown to have beneficial effects when dosed in male PS19 mice from 3 to 6 months of age [25]. As in our prior prevention study with EpoD in PS19 mice [25], we examined MT density and axonal dystrophy in the ON, as well as brain tau pathology and cognitive performance. In addition, complete blood cell counts were obtained to determine whether changes in mitotic blood cells were observed upon prolonged treatment with 51657. We were particularly interested in determining whether compound treatment affected neutrophils, as neutropenia is a primary dose-limiting side-effect observed with MT-stabilizing drugs in cancer patients [57, 58].

**Fig. 2:**
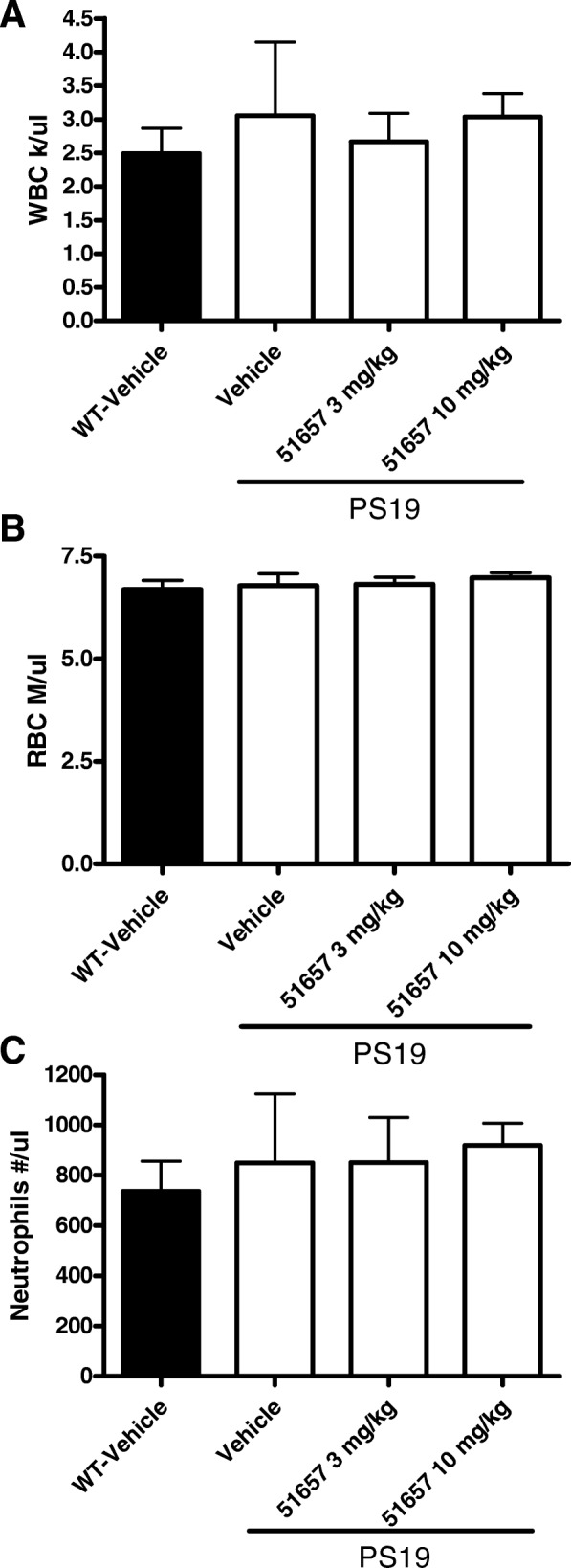
PS19 mouse blood cells were unaffected by 12 weeks of 51657 dosing. Total blood cell counts were determined for WT mice receiving vehicle (*n* = 10) or PS19 mice receiving vehicle (*n* = 6), 3 mg/kg of 51657 (*n* = 7) or 10 mg/kg of 51657 (*n* = 8). No differences in (**a**) total white cell counts, (**b**) red cell counts or **(c)** neutrophil counts were observed between the vehicle- and 51657-treated mice as determined by one-way ANOVA. Error bars represent SEM

The 3-month treatment of PS19 mice with 51657 appeared to be well-tolerated at both doses, as there were no significant changes in body weight between the vehicle- and 51657-treated PS19 mice. None of the treatment groups showed meaningful body weight loss and PS19 mice receiving 51657 showed somewhat less body weight loss than did the vehicle-treated PS19 mice, although this difference did not reach statistical significance (Additional file 1: Figure S3). Similarly, there were no differences in organ weights when normalized to body weight between the vehicle- and 51657-treated mice (Additional file 1: Figure S4). Importantly, there was no evidence of a compound-mediated changes in blood cells, as total white blood cells (Fig. 2a), red blood cells (Fig. 2b) and neutrophils (Fig. 2c) were unchanged in 51657-treated mice relative to either PS19 or WT mice receiving vehicle only.

**Fig. 3:**
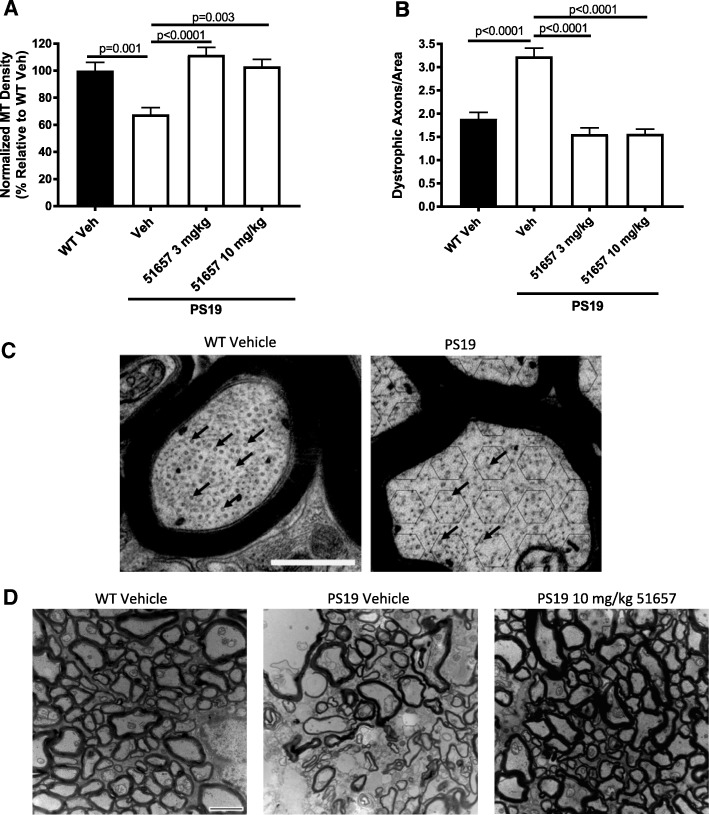
PS19 mice treated with 51657 had significantly increased ON MT density and reduced axonal dystrophy. ON sections from vehicle-treated WT mice (n = 10) and PS19 mice treated with vehicle (*n* = 9) or 3 mg/kg (n = 8) or 10 mg/kg (n = 10) of 51657 were imaged by EM, and the number of MTs and dystrophic axons within treatment-masked images were counted as previously described [25]. **a** Quantification of MT density in ON sections demonstrates that vehicle-treated PS19 mice have a MT deficit relative to vehicle-treated WT mice, and treatment of PS19 mice with 3 mg/kg or 10 mg/kg of 51657 increases MT density to a level comparable to that of WT mice. **b** Quantification of ON EM images reveals a significant reduction in axonal dystrophy in PS19 mice receiving either 3 mg/kg or 10 mg/kg of 51657 compared to vehicle-treated PS19 mice. After quantification, a Grubb’s test determined there was an extreme outlier within the 10 mg/kg 51657 group that was not used for quantification. Analyses consisted of a one-way ANOVA with Tukey’s post-hoc analysis of between group differences. Error bars represent SEM. **c** Representative ON images from a vehicle-treated WT and PS19 mouse, with example MTs indicated by arrows. As depicted in the PS19 vehicle image, hexagonal fields of 0.035 μm^2^ were overlaid on the ON images, with MTs counted within the hexagon and on three of the six borders to avoid repeat counting of MTs of MTs on adjacent hexagons (see also [27]). Scale bar represents 0.5 μM. **d** Representative ON images from a vehicle-treated WT and PS19 mouse, as well as a PS19 mouse that received a twice-weekly dose of 10 mg/kg of 51657. Vehicle-treated PS19 mice have greater axonal dystrophy, as evidenced by fewer intact axons and more axons that are demyelinated or debris-filled, than vehicle-treated WT mice. ONs of PS19 mice treated with 51657 more closely resembled those of vehicle-treated WT mice. Scale bar = 2 μm

To assess whether 51657 improved MT density in the treated PS19 mice, as previously observed with EpoD [25, 27] and dictyostatin [32], ON segments were removed from the study mice after perfusion and sacrifice, and were fixed to allow for EM analysis of MTs in cross-sectional images via blinded quantification. As previously observed in 6-month old male PS19 mice [25], 12-month old vehicle-treated female PS19 mice showed reduced ON MT density relative to age-matched vehicle-treated non-transgenic littermates (Fig. 3a and c). Notably, the PS19 mice receiving either 3 mg/kg or 10 mg/kg of 51657 had a significant increase in MT density that reached the value observed in the WT mice (Fig. 3a). Thus, the twice-weekly dosing scheme with 51657 had the desired effect of abrogating the MT deficit observed in the PS19 mice, with the magnitude of MT enhancement being similar to that previously observed with EpoD [25]. In prior studies with PS19 mice, a reduction of ON MTs coincided with a significant increase in ON axonal dystrophy, with an abundance of swollen and demyelinated axons observed upon EM analysis [25, 27, 32]. An increase in dystrophic axons was also observed in the 12-month old female PS19 mice from the current study, and both doses of 51657 led to a dramatic lowering of ON axonal dystrophy to the level observed in the vehicle-treated WT mice (Fig. 3b and d). These results provide further evidence of 51657 having the desired effect of increasing CNS MTs and improving axonal integrity and function.

**Fig. 4:**
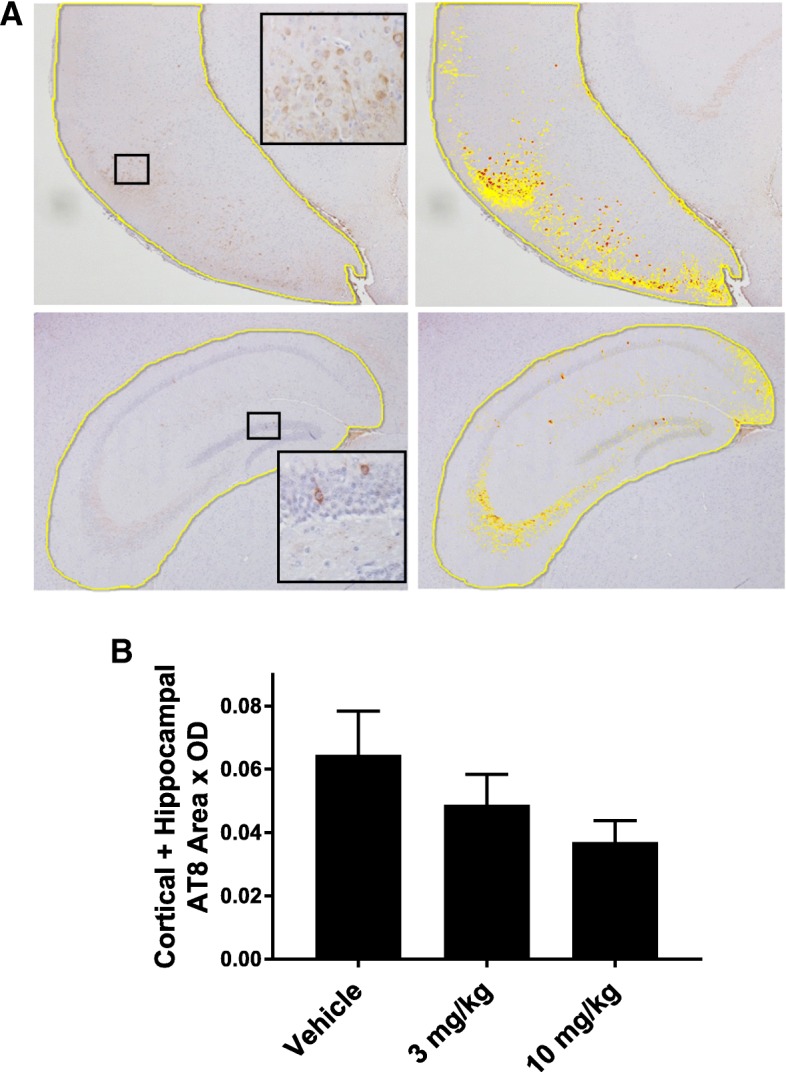
IHC staining and quantification of tau pathology. Three bregma-matched brain sections from each PS19 mice receiving vehicle (*n* = 12), or 3 mg/kg (*n* = 11) or 10 mg/kg (n = 12) of 51657, were stained with AT8 antibody to visualize tau pathology. **a** Representative images from a vehicle-treated PS19 mouse with an average amount of tau pathology (Bregma − 2.5). Regions of stained sections encompassing the hippocampus and entorhinal cortex were imaged and a fixed threshold was applied to distinguish AT8-positive staining from background (images on right; yellow, low AT8 signal; orange, moderate AT8 signal; red, high AT8 signal), followed by quantification of AT8-positive tau pathology. **b** A plot of the combined AT8-positive pathology from the entorhinal cortex and hippocampus from PS19 mice in each treatment group reveals a trend toward reduced tau pathology in the 51657-treated mice

One hemisphere from each brain of the study mice was flash frozen for biochemical measurement of insoluble tau pathology, and the other hemisphere was fixed for immunohistochemical (IHC) evaluation. In our prior prevention study of EpoD in young male PS19 mice, we observed a modest amount of AT8 (pS202/pT205)-positive tau pathology in 6-month old male PS19 mice upon IHC assessment, with a non-significant trend toward reduced pathology in the EpoD-treated mice [25]. A significant reduction of tau pathology was seen in an intervention study in older PS19 mice with greater tau pathology [27]. An examination of AT8-positive tau in the 12-month old female PS19 mice via IHC analysis revealed somewhat less tau pathology than previously observed in 6-month old male PS19 mice, with the amount of AT8 staining being low-to-moderate in vehicle-treated female PS19 mice (Fig. 4a) with significant mouse-to-mouse variability, as previously observed with male PS19 mice [25, 27]. Nonetheless, we attempted to quantify the AT8-positive staining, with analysis of the hippocampus and entorhinal cortex where the majority of tau pathology was observed (Fig. 4a). A blinded assessment of three Bregma-matched sections from each study mouse revealed a non-significant trend toward a reduction of combined cortical and hippocampal AT8-positive staining in the 51657-treated mice (Fig. 4b) that resembled the results previously observed in the prevention study with EpoD in PS19 mice. As previously observed in 6-month old male PS19 mice [25], NeuN staining of neurons revealed no evidence of hippocampal CA3 neuron loss in the female PS19 mice of this study (Additional file 1: Figure S5), a brain region where significant neuron loss is observed in PS19 mice with greater pathology [27]. This is consistent with the relatively modest level of tau pathology observed in these mice.

**Fig. 5:**
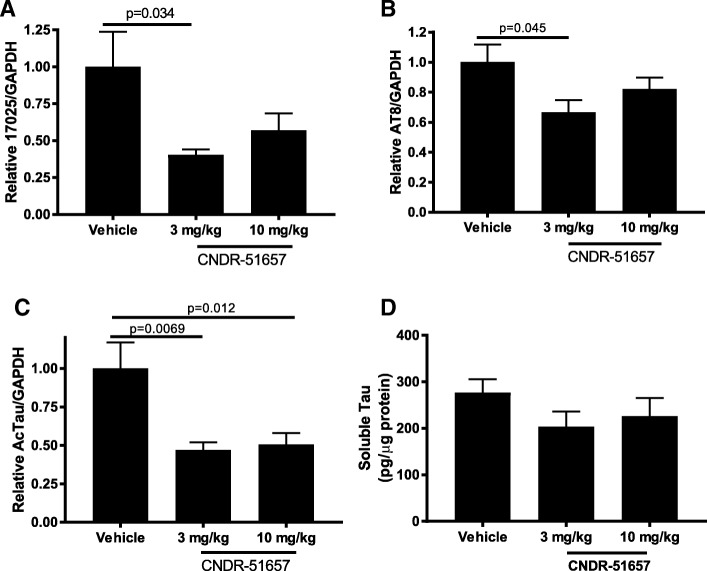
PS19 mice treated with 51657 show a reduction of insoluble pathological forms of tau. Brains of PS19 mice treated with vehicle (*n* = 12), or 3 mg/kg (n = 11) or 10 mg/kg (n = 12) of 51657 were sequentially extracted to remove high salt- and RIPA-soluble proteins. The remaining insoluble fraction was solubilized in 2% SDS and analyzed by immunoblotting, utilizing antibodies that recognize (**a**) total tau (17025 antibody), (**b**) phospho-tau (AT8 antibody) and (**c**) tau acetylated at residue K280 (AcTau; tau K280 antibody). The lower dose of 51657 caused a significant reduction of all three forms of insoluble tau, and the higher dose of 51657 dose resulted in a reduction of all insoluble tau species, with a significant reduction of AcTau. After quantification, a Grubb’s test determined there were extreme outliers within some treatment groups, resulting in the removal of a sample (B2) from the 10 mg/kg 51657 group from the AcTub antibody immunoblot (see Additional file 1: Figure S6), and a sample from each treatment group in the 17025 antibody immunoblot. **d** Soluble tau levels as measured by ELISA within the high salt fractions were unaffected by 51657-treatment. All comparisons consisted on one-way ANOVA with Tukey’s post-hoc analysis of between group differences. Error bars represent SEM

Given the generally low amount of tau pathology observed by AT8-staining in the 12-month old female PS19 mice, a degree of regional variability in the location of AT8-positive tau and the semi-quantitative nature of IHC measurements, we also conducted biochemical assessments of tau pathology since such analyses are generally more quantitative than IHC. The entire cortex and hippocampus from frozen brain hemispheres from each PS19 mouse were subjected to sequential extraction, with homogenization first in high-salt buffer followed by centrifugation, with subsequent extraction of the pellet in RIPA buffer with centrifugation. The remaining high salt- and RIPA-insoluble pellet fraction was solubilized in SDS and analyzed by immunoblotting to determine the amount of total tau, AT8-positive phosphorylated tau, and K280-acetylated tau [44] in the buffer-insoluble fraction. Both AT8 and acetyl-K280 tau have been shown to be enriched in pathological tau, with the latter appearing in more mature tau inclusions [59, 60]. The samples were blinded prior to gel loading and quantification, and the results revealed that the 3 mg/kg dose of 51657 led to a significant reduction of insoluble total tau (Fig. 5a), AT8-positive tau (Fig. 5b) and acetyl-K280-positive tau (Fig. 5c and Additional file 1: Figure S6). The 10 mg/kg dose of 51657 caused a significant reduction of insoluble acetyl-K280 tau (Fig. 5c and Additional file 1: Figure S6), although the observed decrease in insoluble AT8-positive tau and total insoluble tau did not reach statistical significance (Fig. 5a and b). A comparison of the amount of insoluble AcTau in 9-month old female PS19 mice (i.e., start of treatment) to that within 12-month female PS19 mice suggests that there is roughly a doubling of the amount of mature pathological tau over the 3-month treatment period (Additional file 1: Figure S7), and thus an ~ 50% reduction of tau pathology would be expected if 51657 treatment led to a cessation of further pathology development. This is approximately the effect size observed in the 51657-treated PS19 mice (Fig. 5). As the amount of insoluble AT8-positive and total insoluble tau did not differ significantly between the 3 mg/kg and 10 mg/kg 51657 treatment groups, we cannot conclude that the higher dose was less effective than the lower dose, particularly since both doses significantly improved MT density and reduced axonal dystrophy. The reductions in insoluble tau species in the PS19 mice receiving 51657 were not due to an overall reduction in tau protein expression, as soluble tau levels were not significantly different in the vehicle- and 51657-treated PS19 mice (Fig. 5d). An assessment of GFAP-positive astrocytes and Iba1-positive microglia did not reveal noticeable differences in cell density or morphology in any PS19 mouse treatment group (Additional file 1: Figure S8), suggesting that the reduction of tau pathology in the 51657-treated mice was not due to compound-induced effects on glia. In summary, these data reveal that 51657 treatment led to a reduction of insoluble tau pathology in the PS19 mice, as previously demonstrated for EpoD in an interventional study [27] and for which there was a trend toward reduction upon EpoD treatment in a prior prevention study [25]. These changes in tau pathology are believed to result directly from compound-mediated effects on MTs.

**Fig. 6:**
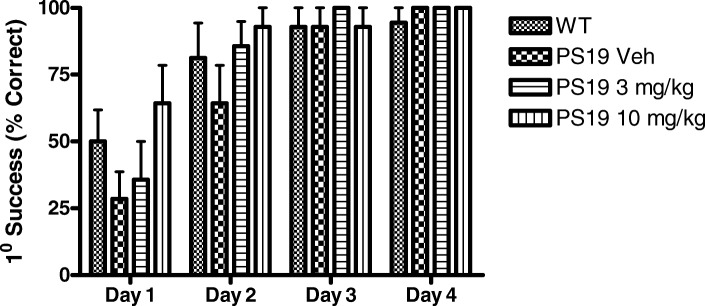
Barnes maze testing of WT and PS19 mice treated with vehicle or 51657. The vehicle-treated PS19 mice had a modest deficit relative to vehicle-treated WT mice in successfully identify the escape compartment in the maze during the first two days of testing, and the PS19 mice receiving 51657 showed a non-significant trend toward improvement on day 1 and 2 compared to the vehicle group. Because of the modest amount of tau pathology and absence of overt neuron loss in the 12-month female PS19 mice, the behavioral deficits were mild and all treatment groups showed nearly 100% performance by the third and fourth days of testing

We have previously observed mild cognitive deficits in male PS19 mice as young as 6 months of age [25]. Thus, the female PS19 mice and littermate controls within this study underwent testing in the Barnes maze shortly after receiving their last vehicle or compound administration. As summarized in Fig. 6, there was a trend towards impaired performance by the vehicle-treated female PS19 mice relative to vehicle-treated non-transgenic littermates during the first two days of testing, as measured by their success in identifying an escape compartment, with the 51657-treated PS19 mice performing somewhat better than the vehicle PS19 group on these days. However, group variability was relatively large and these differences did not reach statistical significance. All treatment groups showed nearly complete learning by days 3 and 4 of testing, which in light of the modest amount of tau pathology and absence of neuron loss in the 12-month old female PS19 is not surprising. Thus, the totality of study data reveal that treatment of female PS19 mice with 51657 from 9- to 12-months of age in a secondary prevention model led to significantly improved MT density and reduced axonal dystrophy, with a resulting reduction of tau pathology and a trend toward improved cognitive performance.

## Discussion

The concept of utilizing MT-stabilizing agents to treat tauopathies has been discussed for some time [31], and our studies and those from others over the past several years have demonstrated the potential of this therapeutic strategy in tauopathy mouse models [24–27, 32, 61, 62], as well as in other tau model systems [63, 64]. Among traditional small molecule MT-stabilizing compounds, both EpoD and the abeotaxane, TPI-287, have progressed to Phase 1b clinical testing, where each was examined in short 2–3 month studies in AD and/or tauopathy patients. Both of these drug candidates appeared to be well tolerated at the tested doses [65]. Interestingly, TPI-287 treatment was reported to result in a significant improvement in AD patient MMSE scores relative to the placebo group after 12 weeks of drug dosing (www.corticebiosciences.com; 11/03/17 press release). However, given the very short duration of these Phase 1b trials and the small number of patients evaluated, we believe caution should be exercised in drawing either positive or negative inferences about the potential of MT-stabilizing agents in neurodegenerative disease from these studies, particularly since disease-modifying trials for AD are typically at least 18–24 months in length.

It is unclear whether either BMS-241027/EpoD or TPI-287 will advance into larger clinical studies of longer duration. Given this and the fact that both of these natural product-derived molecules bind the taxane-site on MTs, we have further investigated the TPD series of small molecule MT-stabilizing molecules which bind to a distinct region on MTs [38], with the goal of identifying alternative and potentially improved candidates for development as disease-modifying drugs for AD and other neurodegenerative diseases. Such molecules have potential advantages over the existing classes of MT-stabilizing natural products, especially in terms of drug-like physicochemical properties and synthetic accessibility. As previously detailed [35], we discovered that the TPDs could be broadly categorized into two distinct groups; those that elicit an undesirable bell-shaped concentration-response profile in in vitro models with an induction of proteasome-mediated tubulin degradation, and a smaller set referred to as TPD+ compounds that are generally characterized by the absence of a *para* alkoxy side-chain on the phenyl group and which exhibit the desired properties of increasing MT stability and MT mass in cellular models [35].

The in vivo characterization of various TPD+ examples revealed that nearly all have excellent brain exposure [35], and we selected 51657 as a prototype for more complete in vivo testing. Although 51657 was found to have a relatively short plasma and brain half-life, the compound caused a significant increase in brain AcTub that could be observed up to 3 days after cessation of dosing. This suggests that MT stabilization persists after most, if not all, of the compound is cleared from the brain. We are unsure of the mechanism of this lasting effect, but perhaps tubulin acetylation or other tubulin post-translational modifications that occur after initial compound-mediated stabilization contribute to prolonged MT activity [39]. Importantly, these data indicate that long brain half-life may not be a necessity for a beneficial MT-stabilizing effect, as was previously suggested based on the long brain retentions of EpoD and dictyostatin [41, 50]. Thus, MT-stabilizing agents with shorter brain half-lives, such as 51657, might provide advantages over the previously examined natural products in that they would still allow for relatively infrequent dosing but with a reduced risk of compound accumulation in the brain and other tissues after repeated dosing.

The ability of 51657 to improve CNS MT density and reduce axonal dystrophy in PS19 mice with twice-weekly dosing further supports the conclusion that a MT-stabilizing compound with a relatively short half-life can be efficacious. The extent of 51657-mediated improvement in MT density in the PS19 tau transgenic mice was comparable to that previously observed with EpoD [25, 27], as was the compound-induced reduction in axonal dystrophy. Moreover, 51657 treatment led to a reduction in insoluble pathologic tau in PS19 mice, as has been observed for both EpoD [24, 25] and dictyostatin [32]. The observations of MT-stabilizing agents reducing tau pathology in tau Tg mice is interesting and arguably somewhat unexpected since compounds that improve MT structure/function would not necessarily be expected to affect the accumulation of misfolded tau. However, it has previously been demonstrated that there is a relationship between impaired axonal transport and tau pathology, as genetically crossing mice with defective kinesin-1 function with tau Tg mice resulted in an exacerbation of tau pathology that may have resulted, at least in part, from JNK pathway activation [66, 67]. Thus, it might be expected that, conversely, normalization of MT function and axonal transport in PS19 mice with a MT-stabilizing agent would lead to a reduction of tau pathology.

Our data reveal that a brain-penetrant MT-stabilizing TPD+ molecule that is readily synthesized can provide meaningful benefit in an established mouse model of tauopathy. Moreover, the lack of effect on dividing blood cells after twice-weekly 51657 administration indicates that axonal MTs can be modulated to provide CNS benefit while avoiding the untoward side-effects observed when high doses of MT-stabilizing drugs are used for the treatment of cancer. Interestingly, the TPD+ molecules described here bind to MTs at a site that is distinct from the binding site of taxanes, epothilones and dictyostatin, with TPD+ molecules interacting at the vinca alkaloid site on MTs [38]. Notably, the binding of vinblastine and vincristine to this site results in MT depolymerization [68], and thus TPD+ compounds are unique in their ability to stabilize MTs through interaction at this site. Moreover, TPD+ molecules appear to promote stability through longitudinal tubulin contacts in MTs, and importantly, do not enhance lateral MT contacts as observed with compounds that bind the taxane site [38]. Thus, 51657 and related TPD+ molecules provide a promising class of brain-penetrant and orally bioavailable MT-stabilizing agents. In addition, 51657 and other TPD molecules generally do not interact with Pgp, unlike EpoD [35] and many taxanes [53, 69]. The absence of Pgp binding provides a potential safety benefit, as this transporter prevents xenobiotics from entering the brain and thus inhibitors of Pgp could increase brain exposure to unwanted molecules. Moreover, the brain and a number of tumors exclude MT-stabilizing drugs through Pgp efflux, and thus higher or more frequent doses of 51657 or other non-Pgp binding TPD molecules might have utility for the treatment of tumors that are resistant to existing MT-directed drugs, particularly brain tumors.

## Conclusions

We demonstrate for the first time that a vinca site-binding TPD+ compound (51657) with favorable drug-like properties is capable of increasing CNS MT stabilization in an established mouse model of tauopathy at a relatively low dose administered twice-weekly, with a resulting reduction of axonal dystrophy and tau pathology in the brain. These beneficial effects are comparable to what we and others have found in mouse tauopathy models with MT-stabilizing natural products like paclitaxel [26], EpoD [24, 25, 27] and dictyostatin [32] that bind the taxane-site on MTs. These results thus reveal that brain-penetrant TPD+ molecules hold considerable promise for the treatment of AD and related tauopathies, as well as possibly additional neurodegenerative disorders [22].

## Additional file

Additional file 1: **Figure S1.** Metabolism of 51657. **Figure S2.** ADR-Res cells expressing Pgp are more sensitive to 51657 than to paclitaxel. **Figure S3.** Normalized WT and PS19 mouse body weights over time while receiving twice-weekly dosing of vehicle or 51657 (3 mg/kg or 10 mg/kg). **Figure S4.** PS19 mouse organ weights were unaffected by 12 weeks of 51657 dosing. **Figure S5.** Quantification of NeuN-positive neurons within the CA3 region of the hippocampus of 12-month old female WT mice or vehicle- or 51657-treated female PS19 mice. **Figure S6.** Composite images of the three blots utilized in quantification of insoluble AcTau as shown in manuscript Fig. 5c. **Figure S7.** A comparison of insoluble AcTau levels in 9-month old and 12-month old female PS19 mice. **Figure S8.** Representative 40× images of hippocampal dentate region from brain sections of vehicle- or 51657-treated PS19 mice stained to visualize astrocytes and microglia. **Table S1.** Crystal data and structure refinement for CNDR-51657. **Table S2.** Atomic coordinates (× 104) and equivalent isotropic displacement parameters (Å2x 103) for CNDR-51657. **Table S3.** Bond lengths [Å] and angles [°] for CNDR-51657. **Table S4.** Anisotropic displacement parameters (Å2x 103) for CNDR-51657. **Table S5.** Hydrogen coordinates (× 104) and isotropic displacement parameters (Å2x 10 3) for CNDR-51657. (PDF 2847 kb)

## Abbreviations

AcTub: Acetyl-tubulin
AD: Alzheimer’s disease
CBD: Corticobasal degeneration
ELISA: Enzyme-linked immunosorbent assay
EM: Electron microscopy
EpoD: Epothilone D
FTLD: Frontotemporal lobar degeneration
GluTub: Detyrosinated COOH-terminal glutamate tubulin
i.p.: Intraperitoneally
IACUC: Institutional animal care and use committee
MT: Microtubule
NFT: Neurofibrillary tangle
NT: Neuropil threads
ON: Optic nerve
PK: Pharmacokinetic
PPD: Phenylpyrimidine
PSP: Progressive supranuclear palsy
Tg: Transgenic
TPD: Triazolopyrimidine
WT: Wild-type
