# Supplementary material for: A brain-penetrant triazolopyrimidine enhances microtubule-stability, reduces axonal dysfunction and decreases tau pathology in a mouse tauopathy model
Source: Mol Neurodegener. 2018 Nov 7;13:59. doi: 10.1186/s13024-018-0291-3 (PMC6223064; doi:10.1186/s13024-018-0291-3)
Supplement: Supplementary file 1 — Figure S1. Metabolism of 51657. Figure S2. ADR-Res cells expressing Pgp are more sensitive to 51657 than to paclitaxel. Figure S3. Normalized WT and PS19 mouse body weights over time while receiving twice-weekly dosing of vehicle or 51657 (3 mg/kg or 10 mg/kg). Figure S4. PS19 mouse organ weights were unaffected by 12 weeks of 51657 dosing. Figure S5. Quantification of NeuN-positive neurons within the CA3 region of the hippocampus of 12-month old female WT mice or vehicle- or 51657-treated female PS19 mice. Figure S6. Composite images of the three blots utilized in quantification of insoluble AcTau as shown in manuscript Fig. 5c. Figure S7. A comparison of insoluble AcTau levels in 9-month old and 12-month old female PS19 mice. Figure S8. Representative 40× images of hippocampal dentate region from brain sections of vehicle- or 51657-treated PS19 mice stained to visualize astrocytes and microglia. Table S1. Crystal data and structure refinement for CNDR-51657. Table S2. Atomic coordinates (× 104) and equivalent isotropic displacement parameters (Å2x 103) for CNDR-51657. Table S3. Bond lengths [Å] and angles [°] for CNDR-51657. Table S4. Anisotropic displacement parameters (Å2x 103) for CNDR-51657. Table S5. Hydrogen coordinates (× 104) and isotropic displacement parameters (Å2x 10 3) for CNDR-51657. (PDF 2847 kb) [file 13024_2018_291_MOESM1_ESM.pdf]

A. Additional Figures

A.

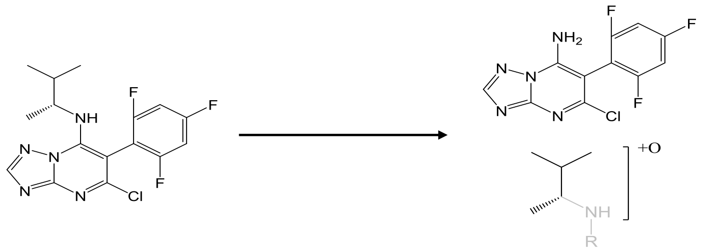

B.

CYP	Inhibitor	Tested at (µM)	RNH_2 Metabolite Formation (% Inhibition)
3A	Ketoconazole	0.5	90 ± 20
2D6	Quinidine	0.5	70 ± 20
2C9	Sulphaphenazole	3	37 ± 7
2C19*	Nootkatone	5	36 ± 4
	Omeprazole	30	80 ± 10

C.

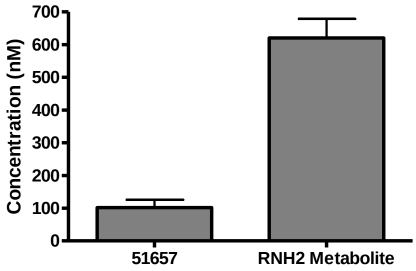

Figure S1. Metabolism of 51657. **A**. In studies with human and mouse microsomes, 51657 is metabolized to yield the N-dealkylated TPD as a primary metabolite that is devoid of MT-stabilizing activity. **B**. Inhibitors of multiple CYP450 isozymes slow the metabolism of 51657, revealing that multiple CYP450 isozymes contribute to the production of the N-dealkylated metabolite. Values represent % inhibition and SD of conversion of the parent molecule to primary metabolite based on 3 independent incubations with each inhibitor. **C**. Levels of 51657 and the N-dealkylated metabolite in the brains of WT mice (n=3) 4 h after i.p. administration of 5 mg/kg of 51657. Error bars represent SEM.

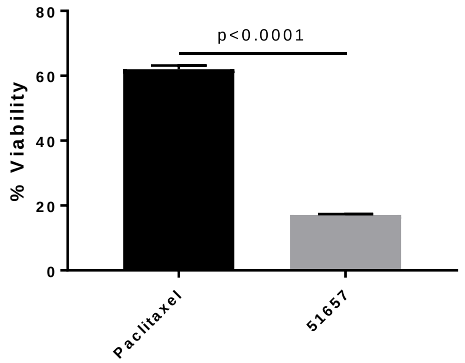

Figure S2. ADR-Res cells expressing Pgp are more sensitive to 51657 than to paclitaxel. Wells of dividing ADR-Res cells (n=8) were treated with either 1 μM paclitaxel, 1 μM 51657 or vehicle, and cells were examined for viability 72 h after treatment. The difference between the paclitaxel and 51657 treatment groups was highly significant ($p<0.0001$) as determined by an unpaired t-test. Error bars represent SEM.

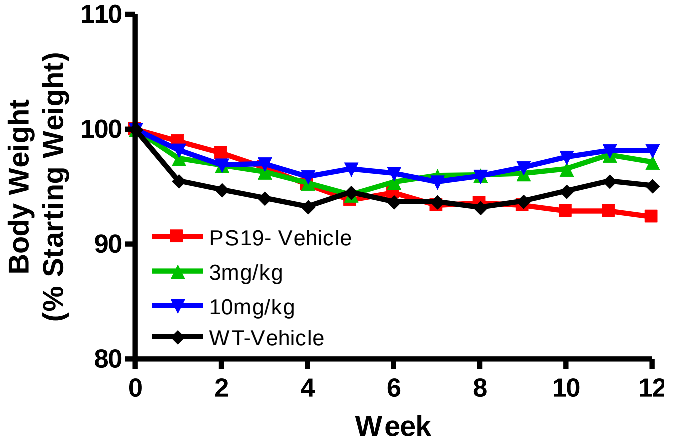

Figure S3. Normalized WT and PS19 mouse body weights over time while receiving twice-weekly dosing of vehicle or 51657 (3 mg/kg or 10 mg/kg). Mice were 9 months of age at study initiation, and body weights were normalized to the starting body weight for each mouse, with the mean normalized weight for each study group (n=12) plotted as a function of time after initiation of dosing (error bars not shown). PS19 mice receiving 51657 showed a trend toward less body weight loss than vehicle-treated PS19 mice, although this did not reach statistical significance as determined by two-way ANOVA comparing body weights vs. time.

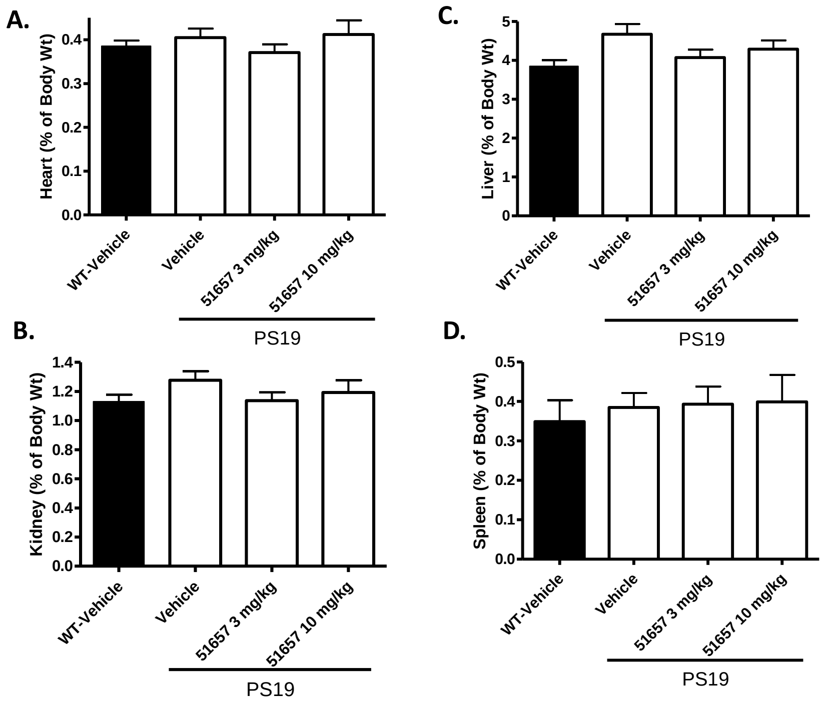

Figure S4. PS19 mouse organ weights were unaffected by 12 weeks of 51657 dosing. Organ weights of vehicle- or 51657-treated WT and PS19 mice (n=12/group) were normalized to mouse body weight, and there were no significant differences in **A**. heart, **B**. kidney, **C**. liver or **D**. spleen weights between any of the treatment groups as determined by one-way ANOVA. Error bars represent SEM.

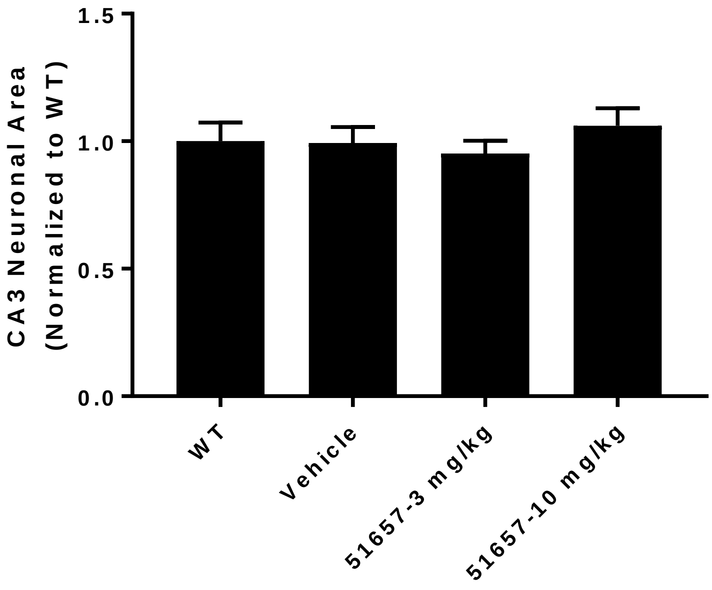

Figure S5. Quantification of NeuN-positive neurons within the CA3 region of the hippocampus of 12-month old female WT mice or vehicle- or 51657-treated female PS19 mice. No significant differences in NeuN-positive area were observed among the treatment groups (one-way ANOVA).

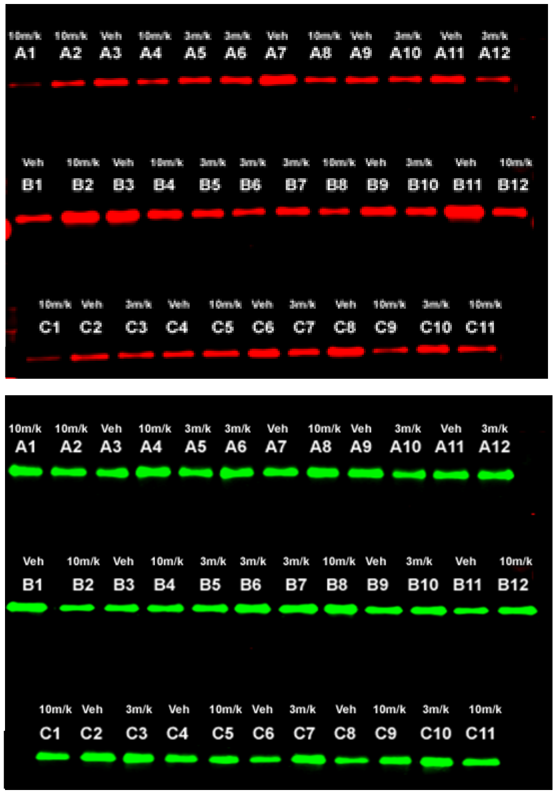

Figure S6. Composite images of the three blots utilized in quantification of insoluble AcTau as shown in manuscript Fig. 5C. The AcTau blots (top) and GAPDH blots (bottom) were masked prior to sample loading and quantification.

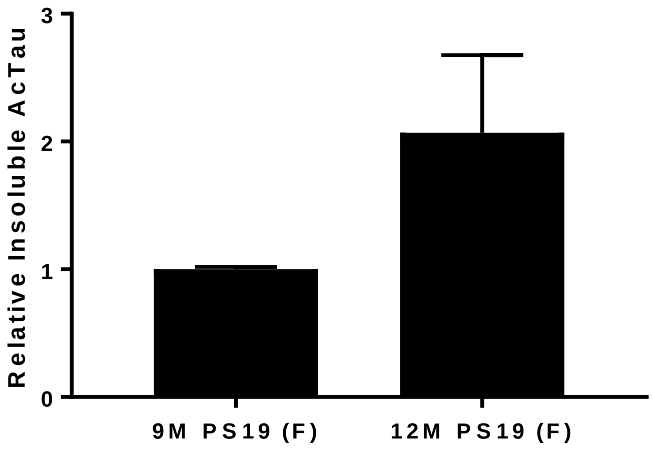

Figure S7. A comparison of insoluble AcTau levels in 9-month old and 12-month old female PS19 mice (n=3 at each age), as determined by immunoblotting. Error bars are SEM.

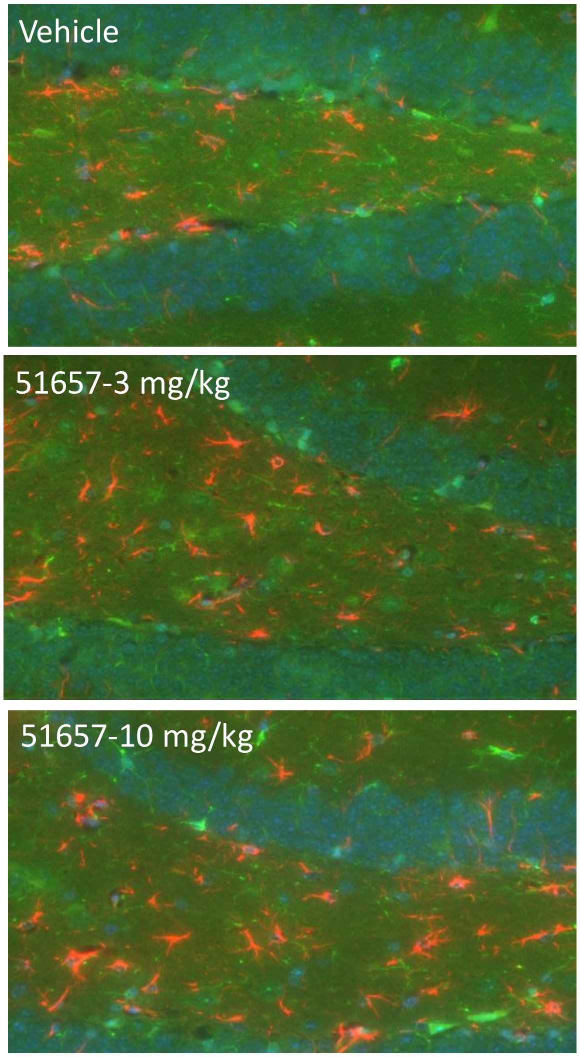

Figure S8. Representative 40X images of hippocampal dentate region from brain sections of vehicle- or 51657-treated PS19 mice stained to visualize astrocytes (GFAP; red fluorescence) and microglia (Iba1; green fluorescence). No notable differences in astrocyte or microglial cell density or cellular morphology were noted in stained sections from multiple brain regions in PS19 mice (n=3) from each treatment group.

B. X-Ray Data for 51657

December 6, 2017 **Subject: CNDR-51657**

Crystal Structure Report

For The Ballatore Lab @ UCSD

Dr. Milan Gembicky

UCSD Crystallography Lab

5128 Urey Hall

9500 Gilman Dr

La Jolla, CA 92093-0358

Tel: (858) 822-3871

Fax: (858) 822-3872

mgembicky@ucsd.edu

Experimental Summary

The single crystal X-ray diffraction studies were carried out on a Bruker X8 Apex II CCD diffractometer equipped with Mo K_α radiation (λ = 0.71073). A 0.250 x 0.220 x 0.200 mm colorless crystal was mounted on a Cryoloop with Paratone oil.

Data were collected in a nitrogen gas stream at 100(2) K using ϕ and ϖ scans. Crystal-to-detector distance was 40 mm using variable exposure time 8s with a scan width of 0.8°. Data collection was 100% complete to 25.242° in θ.

A total of 27448 reflections were collected covering the indices, -9<=h<=9, -19<=k<19, -18<=l<=18. 7403 reflections were found to be symmetry independent, with a R_{int} of 0.0250. Indexing and unit cell refinement indicated a primitive, **Monoclinic** lattice. The space group was found to be $\boldsymbol{P2_1}$ The data were integrated using the Bruker SAINT software program and scaled using the SADABS software program. Solution by direct methods (SHELXT) produced a complete phasing model consistent with the proposed structure.

All nonhydrogen atoms were refined anisotropically by full-matrix least-squares (SHELXL-2014). All carbon bonded hydrogen atoms were placed using a riding model. Their positions were constrained relative to their parent atom using the appropriate HFIX command in SHELXL-2014. All other hydrogen atoms (N- H) were located in the difference map (N-H, O-H). There positions were freely refined with their thermal parameter refined using "riding" model. Crystallographic data are summarized in Table S1.

Notes: Proposed structure agrees with model, two independent molecules in asymmetric unit.
Excellent data and refinement
Absolute stereochemistry "R" please check
Absolute structure parameter 0.002(19) conclusive

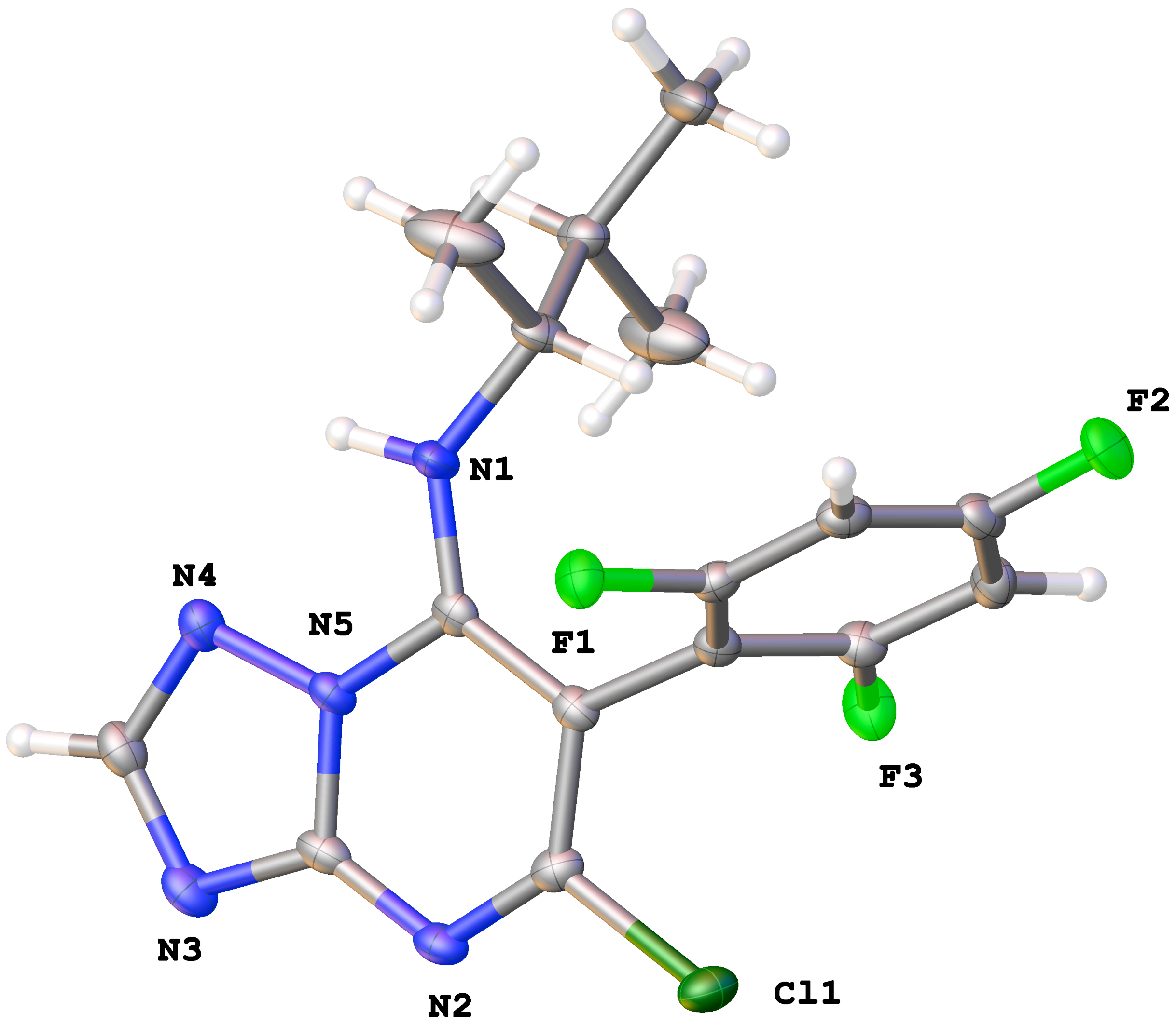

F2
N1
N4
N5
F1
F3
N3
N2
Cl1

Table S1. Crystal data and structure refinement for CNDR-51657.

Report date	2017-12-06	
Identification code	ballatore02	
Empirical formula	C16 H15 Cl F3 N5	
Molecular formula	C16 H15 Cl F3 N5	
Formula weight	369.78	
Temperature	100.0 K	
Wavelength	0.71073 Å	
Crystal system	Monoclinic	
Space group	P 1 21 1	
Unit cell dimensions	a = 7.3945(3) Å	α= 90°.
	b = 15.3691(7) Å	β= 94.243(2)°.
	c = 14.7831(7) Å	γ = 90°.
Volume	1675.45(13) Å^3	
Z	4	
Density (calculated)	1.466 Mg/m^3	
Absorption coefficient	0.268 mm^{-1}	
F(000)	760	
Crystal size	0.25 x 0.22 x 0.2 mm^3	
Theta range for data collection	1.381 to 27.110°.	
Index ranges	-9<=h<=9, -19<=k<=19, -18<=l<=18	
Reflections collected	27448	
Independent reflections	7403 [R(int) = 0.0250]	
Completeness to theta = 25.242°	100.0 %	
Absorption correction	Semi-empirical from equivalents	
Max. and min. transmission	0.4912 and 0.4623	
Refinement method	Full-matrix least-squares on F^2	
Data / restraints / parameters	7403 / 1 / 463	
Goodness-of-fit on F^2	1.025	
Final R indices [I>2sigma(I)]	R1 = 0.0300, wR2 = 0.0683	
R indices (all data)	R1 = 0.0367, wR2 = 0.0715	
Absolute structure parameter	0.002(19)	
Largest diff. peak and hole	0.237 and -0.218 e.Å^{-3}	

Table S2. Atomic coordinates (x 10^4) and equivalent isotropic displacement parameters (Å^{2}x 10^3) for CNDR-51657. U(eq) is defined as one third of the trace of the orthogonalized U^{ij} tensor.

	x	y	z	U(eq)
Cl(1)	6955(1)	2219(1)	1678(1)	25(1)
F(1)	11424(2)	2808(1)	39(1)	22(1)
F(2)	7392(2)	2082(1)	-2448(1)	27(1)
F(3)	5238(2)	3452(1)	82(1)	25(1)
N(1)	10258(3)	4940(2)	509(2)	19(1)
N(2)	8308(3)	3498(2)	2630(2)	19(1)
N(3)	9664(3)	4716(2)	3469(2)	21(1)
N(4)	10660(3)	5441(2)	2246(2)	18(1)
N(5)	9819(3)	4679(2)	1985(1)	15(1)
C(1)	10520(4)	5413(2)	3138(2)	20(1)
C(2)	9214(3)	4255(2)	2724(2)	18(1)
C(3)	8111(3)	3210(2)	1793(2)	17(1)
C(4)	8693(3)	3591(2)	997(2)	16(1)
C(5)	9583(3)	4396(2)	1101(2)	15(1)
C(6)	10212(4)	4922(2)	-489(2)	20(1)
C(7)	12183(5)	4939(3)	-740(2)	45(1)
C(8)	9099(3)	5685(2)	-890(2)	20(1)
C(9)	7162(4)	5660(3)	-603(2)	45(1)
C(10)	9078(4)	5675(2)	-1926(2)	34(1)
C(11)	8351(4)	3156(2)	102(2)	16(1)
C(12)	6625(3)	3107(2)	-342(2)	18(1)
C(13)	6249(3)	2742(2)	-1183(2)	21(1)
C(14)	7710(4)	2408(2)	-1603(2)	21(1)
C(15)	9465(4)	2399(2)	-1208(2)	19(1)
C(16)	9719(3)	2779(2)	-358(2)	16(1)
Cl(1')	8343(1)	7725(1)	3315(1)	25(1)
F(1')	9922(2)	6548(1)	4978(1)	25(1)
F(2')	7545(2)	7916(1)	7451(1)	28(1)
F(3')	3707(2)	7172(1)	4897(1)	25(1)
N(1')	4981(3)	5064(2)	4542(2)	19(1)
N(2')	6792(3)	6491(2)	2363(2)	20(1)

N(3')	5358(3)	5276(2)	1547(2)	22(1)
N(4')	4436(3)	4548(2)	2785(2)	19(1)
N(5')	5283(3)	5311(2)	3034(2)	16(1)
C(1')	4530(4)	4579(2)	1891(2)	21(1)
C(2')	5852(3)	5745(2)	2288(2)	18(1)
C(3')	7072(3)	6771(2)	3204(2)	17(1)
C(4')	6504(3)	6393(2)	4007(2)	15(1)
C(5')	5608(3)	5595(2)	3914(2)	15(1)
C(6')	5698(3)	4978(2)	5497(2)	18(1)
C(7')	7515(4)	4506(2)	5536(2)	29(1)
C(8')	4260(3)	4529(2)	6026(2)	19(1)
C(9')	2496(4)	5034(3)	5968(2)	34(1)
C(10')	4950(4)	4408(2)	7018(2)	30(1)
C(11')	6801(4)	6835(2)	4896(2)	16(1)
C(12')	5382(3)	7207(2)	5332(2)	18(1)
C(13')	5567(4)	7587(2)	6180(2)	20(1)
C(14')	7293(4)	7591(2)	6603(2)	20(1)
C(15')	8790(4)	7262(2)	6215(2)	20(1)
C(16')	8490(4)	6893(2)	5365(2)	19(1)

Table S3. Bond lengths [Å] and angles [°] for CNDR-51657.

Cl(1)-C(3)	1.748(3)
F(1)-C(16)	1.351(3)
F(2)-C(14)	1.351(3)
F(3)-C(12)	1.350(3)
N(1)-H(1)	0.92(3)
N(1)-C(5)	1.334(4)
N(1)-C(6)	1.474(3)
N(2)-C(2)	1.345(4)
N(2)-C(3)	1.312(4)
N(3)-C(1)	1.355(4)
N(3)-C(2)	1.331(4)
N(4)-N(5)	1.368(3)
N(4)-C(1)	1.331(3)
N(5)-C(2)	1.375(3)
N(5)-C(5)	1.376(3)
C(1)-H(1A)	0.9500
C(3)-C(4)	1.410(4)
C(4)-C(5)	1.404(4)
C(4)-C(11)	1.488(4)
C(6)-H(6)	1.0000
C(6)-C(7)	1.530(4)
C(6)-C(8)	1.527(4)
C(7)-H(7A)	0.9800
C(7)-H(7B)	0.9800
C(7)-H(7C)	0.9800
C(8)-H(8)	1.0000
C(8)-C(9)	1.524(4)
C(8)-C(10)	1.530(4)
C(9)-H(9A)	0.9800
C(9)-H(9B)	0.9800
C(9)-H(9C)	0.9800
C(10)-H(10A)	0.9800
C(10)-H(10B)	0.9800
C(10)-H(10C)	0.9800
C(11)-C(12)	1.394(4)
C(11)-C(16)	1.386(3)
C(12)-C(13)	1.372(4)
C(13)-H(13)	0.9500
C(13)-C(14)	1.384(4)
C(14)-C(15)	1.383(4)
C(15)-H(15)	0.9500
C(15)-C(16)	1.386(4)
Cl(1')-C(3')	1.743(3)
F(1')-C(16')	1.349(3)
F(2')-C(14')	1.350(3)
F(3')-C(12')	1.354(3)
N(1')-H(1')	0.87(4)
N(1')-C(5')	1.343(4)
N(1')-C(6')	1.477(3)
N(2')-C(2')	1.341(4)
N(2')-C(3')	1.317(4)
N(3')-C(1')	1.351(4)
N(3')-C(2')	1.340(4)
N(4')-N(5')	1.368(3)
N(4')-C(1')	1.329(4)
N(5')-C(2')	1.381(3)
N(5')-C(5')	1.376(3)
C(1')-H(1'A)	0.9500
C(3')-C(4')	1.414(4)
C(4')-C(5')	1.397(4)
C(4')-C(11')	1.481(4)
C(6')-H(6')	1.0000
C(6')-C(7')	1.524(4)
C(6')-C(8')	1.531(3)
C(7')-H(7'A)	0.9800
C(7')-H(7'B)	0.9800
C(7')-H(7'C)	0.9800
C(8')-H(8')	1.0000
C(8')-C(9')	1.515(4)
C(8')-C(10')	1.528(3)
C(9')-H(9'A)	0.9800
C(9')-H(9'B)	0.9800
C(9')-H(9'C)	0.9800
C(10')-H(10D)	0.9800
C(10')-H(10E)	0.9800
C(10')-H(10F)	0.9800
C(11')-C(12')	1.394(4)
C(11')-C(16')	1.386(4)
C(12')-C(13')	1.381(4)
C(13')-H(13')	0.9500
C(13')-C(14')	1.379(4)
C(14')-C(15')	1.380(4)
C(15')-H(15')	0.9500
C(15')-C(16')	1.380(4)
C(5)-N(1)-H(1)	111(2)
C(5)-N(1)-C(6)	131.5(3)
C(6)-N(1)-H(1)	117(2)
C(3)-N(2)-C(2)	114.2(2)
C(2)-N(3)-C(1)	102.5(2)
C(1)-N(4)-N(5)	100.7(2)
N(4)-N(5)-C(2)	110.4(2)

N(4)-N(5)-C(5)	124.2(2)	C(16)-C(11)-C(12)	115.0(3)
C(2)-N(5)-C(5)	125.4(3)	F(3)-C(12)-C(11)	117.5(2)
N(3)-C(1)-H(1A)	121.4	F(3)-C(12)-C(13)	118.1(2)
N(4)-C(1)-N(3)	117.1(3)	C(13)-C(12)-C(11)	124.4(2)
N(4)-C(1)-H(1A)	121.4	C(12)-C(13)-H(13)	121.8
N(2)-C(2)-N(5)	121.1(3)	C(12)-C(13)-C(14)	116.4(2)
N(3)-C(2)-N(2)	129.7(2)	C(14)-C(13)-H(13)	121.8
N(3)-C(2)-N(5)	109.2(3)	F(2)-C(14)-C(13)	117.7(2)
N(2)-C(3)-Cl(1)	113.9(2)	F(2)-C(14)-C(15)	118.7(2)
N(2)-C(3)-C(4)	128.9(3)	C(15)-C(14)-C(13)	123.6(3)
C(4)-C(3)-Cl(1)	117.3(2)	C(14)-C(15)-H(15)	122.0
C(3)-C(4)-C(11)	120.9(3)	C(14)-C(15)-C(16)	116.0(2)
C(5)-C(4)-C(3)	116.4(3)	C(16)-C(15)-H(15)	122.0
C(5)-C(4)-C(11)	122.7(2)	F(1)-C(16)-C(11)	117.7(2)
N(1)-C(5)-N(5)	113.4(3)	F(1)-C(16)-C(15)	117.8(2)
N(1)-C(5)-C(4)	132.5(3)	C(15)-C(16)-C(11)	124.4(3)
N(5)-C(5)-C(4)	114.1(2)	C(5')-N(1')-H(1')	113(2)
N(1)-C(6)-H(6)	108.9	C(5')-N(1')-C(6')	126.7(2)
N(1)-C(6)-C(7)	106.9(2)	C(6')-N(1')-H(1')	116(2)
N(1)-C(6)-C(8)	110.3(2)	C(3')-N(2')-C(2')	113.8(2)
C(7)-C(6)-H(6)	108.9	C(2')-N(3')-C(1')	102.8(2)
C(8)-C(6)-H(6)	108.9	C(1')-N(4')-N(5')	100.3(2)
C(8)-C(6)-C(7)	112.8(2)	N(4')-N(5')-C(2')	111.1(2)
C(6)-C(7)-H(7A)	109.5	N(4')-N(5')-C(5')	124.8(2)
C(6)-C(7)-H(7B)	109.5	C(5')-N(5')-C(2')	124.1(3)
C(6)-C(7)-H(7C)	109.5	N(3')-C(1')-H(1'A)	121.2
H(7A)-C(7)-H(7B)	109.5	N(4')-C(1')-N(3')	117.6(3)
H(7A)-C(7)-H(7C)	109.5	N(4')-C(1')-H(1'A)	121.2
H(7B)-C(7)-H(7C)	109.5	N(2')-C(2')-N(5')	122.2(2)
C(6)-C(8)-H(8)	108.5	N(3')-C(2')-N(2')	129.6(2)
C(6)-C(8)-C(10)	110.3(2)	N(3')-C(2')-N(5')	108.2(3)
C(9)-C(8)-C(6)	111.3(2)	N(2')-C(3')-Cl(1')	114.3(2)
C(9)-C(8)-H(8)	108.5	N(2')-C(3')-C(4')	128.5(3)
C(9)-C(8)-C(10)	109.8(2)	C(4')-C(3')-Cl(1')	117.2(2)
C(10)-C(8)-H(8)	108.5	C(3')-C(4')-C(11')	121.6(3)
C(8)-C(9)-H(9A)	109.5	C(5')-C(4')-C(3')	116.5(3)
C(8)-C(9)-H(9B)	109.5	C(5')-C(4')-C(11')	121.9(2)
C(8)-C(9)-H(9C)	109.5	N(1')-C(5')-N(5')	114.6(3)
H(9A)-C(9)-H(9B)	109.5	N(1')-C(5')-C(4')	130.6(3)
H(9A)-C(9)-H(9C)	109.5	N(5')-C(5')-C(4')	114.8(2)
H(9B)-C(9)-H(9C)	109.5	N(1')-C(6')-H(6')	108.2
C(8)-C(10)-H(10A)	109.5	N(1')-C(6')-C(7')	109.4(2)
C(8)-C(10)-H(10B)	109.5	N(1')-C(6')-C(8')	108.4(2)
C(8)-C(10)-H(10C)	109.5	C(7')-C(6')-H(6')	108.2
H(10A)-C(10)-H(10B)	109.5	C(7')-C(6')-C(8')	114.2(2)
H(10A)-C(10)-H(10C)	109.5	C(8')-C(6')-H(6')	108.2
H(10B)-C(10)-H(10C)	109.5	C(6')-C(7')-H(7'A)	109.5
C(12)-C(11)-C(4)	122.2(2)	C(6')-C(7')-H(7'B)	109.5
C(16)-C(11)-C(4)	122.8(2)	C(6')-C(7')-H(7'C)	109.5

H(7'A)-C(7')-H(7'B)	109.5	H(10E)-C(10')-H(10F)	109.5
H(7'A)-C(7')-H(7'C)	109.5	C(12')-C(11')-C(4')	122.1(2)
H(7'B)-C(7')-H(7'C)	109.5	C(16')-C(11')-C(4')	122.9(2)
C(6')-C(8')-H(8')	108.2	C(16')-C(11')-C(12')	115.0(3)
C(9')-C(8')-C(6')	111.6(2)	F(3')-C(12')-C(11')	117.3(2)
C(9')-C(8')-H(8')	108.2	F(3')-C(12')-C(13')	118.2(2)
C(9')-C(8')-C(10')	110.0(2)	C(13')-C(12')-C(11')	124.5(3)
C(10')-C(8')-C(6')	110.5(2)	C(12')-C(13')-H(13')	122.0
C(10')-C(8')-H(8')	108.2	C(14')-C(13')-C(12')	116.1(2)
C(8')-C(9')-H(9'A)	109.5	C(14')-C(13')-H(13')	122.0
C(8')-C(9')-H(9'B)	109.5	F(2')-C(14')-C(13')	118.8(2)
C(8')-C(9')-H(9'C)	109.5	F(2')-C(14')-C(15')	117.5(2)
H(9'A)-C(9')-H(9'B)	109.5	C(13')-C(14')-C(15')	123.7(3)
H(9'A)-C(9')-H(9'C)	109.5	C(14')-C(15')-H(15')	121.7
H(9'B)-C(9')-H(9'C)	109.5	C(14')-C(15')-C(16')	116.6(2)
C(8')-C(10')-H(10D)	109.5	C(16')-C(15')-H(15')	121.7
C(8')-C(10')-H(10E)	109.5	F(1')-C(16')-C(11')	118.0(3)
C(8')-C(10')-H(10F)	109.5	F(1')-C(16')-C(15')	118.0(2)
H(10D)-C(10')-H(10E)	109.5	C(15')-C(16')-C(11')	124.1(2)
H(10D)-C(10')-H(10F)	109.5		

Table S4. Anisotropic displacement parameters ($Å^2 x 10^3$) for CNDR-51657. The anisotropic displacement factor exponent takes the form: $-2\pi^2[h^2 a^{*2}U^{11} + ... + 2 h k a^* b^* U^{12}]$

	U^{11}	U^{22}	U^{33}	U^{23}	U^{13}	U^{12}
Cl(1)	27(1)	22(1)	25(1)	2(1)	9(1)	-4(1)
F(1)	14(1)	28(1)	24(1)	-4(1)	2(1)	3(1)
F(2)	33(1)	31(1)	17(1)	-7(1)	0(1)	2(1)
F(3)	15(1)	35(1)	25(1)	-8(1)	3(1)	7(1)
N(1)	25(1)	21(1)	12(1)	2(1)	1(1)	-4(1)
N(2)	17(1)	24(1)	15(1)	4(1)	5(1)	4(1)
N(3)	22(1)	27(2)	16(1)	-3(1)	4(1)	8(1)
N(4)	18(1)	19(1)	18(1)	-2(1)	0(1)	4(1)
N(5)	16(1)	18(1)	12(1)	1(1)	2(1)	5(1)
C(1)	19(1)	25(2)	17(1)	-5(1)	1(1)	6(1)
C(2)	14(1)	25(2)	14(1)	4(1)	4(1)	8(1)
C(3)	13(1)	17(2)	22(2)	2(1)	5(1)	3(1)
C(4)	12(1)	20(2)	15(1)	0(1)	3(1)	5(1)
C(5)	12(1)	18(2)	16(1)	2(1)	0(1)	6(1)
C(6)	28(1)	21(1)	13(1)	2(1)	7(1)	2(1)
C(7)	37(2)	61(2)	39(2)	23(2)	20(1)	25(2)
C(8)	18(1)	24(1)	19(1)	5(1)	2(1)	0(1)
C(9)	25(2)	72(3)	41(2)	25(2)	12(1)	13(2)
C(10)	30(2)	49(2)	21(1)	14(1)	-3(1)	-10(2)
C(11)	17(1)	14(2)	16(1)	1(1)	2(1)	2(1)
C(12)	17(1)	20(2)	19(1)	0(1)	5(1)	4(1)
C(13)	17(1)	22(2)	22(1)	1(1)	-2(1)	0(1)
C(14)	27(1)	20(2)	14(1)	-1(1)	2(1)	0(1)
C(15)	21(1)	17(2)	19(1)	1(1)	7(1)	2(1)
C(16)	15(1)	16(2)	19(1)	2(1)	4(1)	0(1)
Cl(1')	31(1)	20(1)	26(1)	3(1)	14(1)	-1(1)
F(1')	16(1)	32(1)	28(1)	-5(1)	5(1)	4(1)
F(2')	34(1)	33(1)	17(1)	-7(1)	1(1)	2(1)
F(3')	14(1)	35(1)	25(1)	-5(1)	3(1)	4(1)
N(1')	21(1)	22(1)	14(1)	1(1)	0(1)	-6(1)
N(2')	18(1)	26(1)	17(1)	4(1)	6(1)	6(1)

N(3')	19(1)	34(2)	14(1)	0(1)	1(1)	7(1)
N(4')	19(1)	22(2)	16(1)	-4(1)	0(1)	3(1)
N(5')	14(1)	19(1)	14(1)	1(1)	1(1)	3(1)
C(1')	19(1)	30(2)	16(1)	-4(1)	0(1)	10(1)
C(2')	15(1)	26(2)	14(1)	3(1)	4(1)	9(1)
C(3')	14(1)	20(2)	18(1)	4(1)	6(1)	7(1)
C(4')	13(1)	16(2)	16(1)	2(1)	3(1)	4(1)
C(5')	14(1)	22(2)	10(1)	1(1)	2(1)	6(1)
C(6')	18(1)	24(1)	11(1)	2(1)	1(1)	-3(1)
C(7')	19(1)	48(2)	21(1)	8(1)	2(1)	1(1)
C(8')	17(1)	23(1)	16(1)	1(1)	3(1)	-3(1)
C(9')	22(1)	47(2)	35(2)	10(2)	9(1)	7(2)
C(10')	24(2)	47(2)	17(1)	8(1)	2(1)	-10(1)
C(11')	19(1)	15(2)	14(1)	2(1)	4(1)	-1(1)
C(12')	16(1)	19(2)	19(1)	2(1)	2(1)	-3(1)
C(13')	22(1)	20(2)	20(1)	-1(1)	8(1)	2(1)
C(14')	29(1)	16(2)	16(1)	-2(1)	2(1)	-2(1)
C(15')	20(1)	19(2)	22(1)	-2(1)	-2(1)	-2(1)
C(16')	17(1)	17(2)	24(2)	1(1)	5(1)	3(1)

Table S5. Hydrogen coordinates (x 10^4) and isotropic displacement parameters ($Å^2$x 10^3) for CNDR-51657.

	x	y	z	U(eq)
H(1)	10770(40)	5420(20)	800(20)	29
H(1A)	10999	5864	3525	24
H(6)	9637	4366	-713	24
H(7A)	12755	5484	-526	67
H(7B)	12214	4899	-1400	67
H(7C)	12841	4446	-454	67
H(8)	9680	6238	-663	25
H(9A)	6573	5120	-822	68
H(9B)	6487	6160	-864	68
H(9C)	7181	5683	60	68
H(10A)	10278	5839	-2111	50
H(10B)	8173	6091	-2180	50
H(10C)	8769	5090	-2151	50
H(13)	5050	2720	-1461	25
H(15)	10438	2147	-1502	22
H(1')	4440(40)	4610(30)	4310(20)	29
H(1'A)	4034	4129	1509	26
H(6')	5905	5575	5752	21
H(7'A)	7325	3901	5342	44
H(7'B)	8069	4518	6159	44
H(7'C)	8319	4796	5133	44
H(8')	4019	3940	5754	22
H(9'A)	2714	5622	6208	51
H(9'B)	1616	4736	6324	51
H(9'C)	2015	5070	5333	51
H(10D)	6012	4024	7053	44
H(10E)	3992	4148	7354	44
H(10F)	5289	4975	7283	44
H(13')	4564	7831	6456	24
H(15')	9968	7288	6517	24
